# High and intermittent hydrostatic pressure promotes maturation of engineered cardiac tissues derived from human pluripotent stem cells

**DOI:** 10.1016/j.stemcr.2026.103019

**Published:** 2026-07-23

**Authors:** Wusiman Maihemuti, Kozue Murata, Takahiro Jimba, Jun Iida, Keisuke Hakamada, Akane Sakaguchi, Mosha Abulaiti, Yuichi Saito, Laura Yuriko González-Teshima, Satoshi Takatori, Takahiro Kenmotsu, Kenichi Yoshikawa, Kenji Minatoya, Seitaro Nomura, Wataru Kimura, Hidetoshi Masumoto

**Affiliations:** 1 Clinical Translational Research Program, RIKEN Center for Biosystems Dynamics Research, 2-2-3 Minatojimaminami-cho, Chuo-ku, Kobe 650-0047, Japan; 2 Department of Cardiovascular Surgery, Graduate School of Medicine, Kyoto University, 54 Kawara-cho, Shogoin, Sakyo-ku, Kyoto 606-8507, Japan; 3 Department of Cardiovascular Medicine, Graduate School of Medicine, The University of Tokyo, 7-3-1, Hongo, Bunkyo-ku, Tokyo 113-0033, Japan; 4 Laboratory for Heart Regeneration, RIKEN Center for Biosystems Dynamics Research, 2-2-3 Minatojimaminami-cho, Chuo-ku, Kobe 650-0047, Japan; 5 Faculty of Life and Medical Sciences, Doshisha University, 1-3 Tatara Miyakodani, Kyotanabe, Kyoto 610-0394, Japan

**Keywords:** engineered cardiac tissues, human pluripotent stem cells, hydrostatic pressure, cardiac maturation, cardiomyocytes, endothelial cells, mechanotransduction, cardiac regeneration

## Abstract

The immaturity of human pluripotent stem cell-derived cardiomyocytes (hPSC-CMs) and engineered cardiac tissues (ECTs) limits their use in regenerative therapies. Ventricular loading pressure plays a vital role in cardiac repair, prompting the hypothesis that hydrostatic pressure could enhance ECT maturation. ECTs were created by co-culturing hPSC-derived cardiovascular cells with extracellular matrix proteins and subjecting them to intermittent hydrostatic pressure (1 h/day at 50 kPa for 3 days after 2 weeks of pre-culture). This protocol improved cardiomyocyte alignment, mitochondrial content, and metabolic and functional maturation, evidenced by enhanced contractility, calcium flux, and single-cell RNA sequencing. Endothelial cells were critical for these effects, as their absence prevented maturation. Hydrostatic pressure combined with dynamic culture also promoted vascular network formation. Transplanted trained ECTs significantly improved ejection fraction in a rat myocardial infarction model. These findings demonstrate the potential of hydrostatic pressure training to advance ECT maturation and therapeutic application.

## Highlights

• Hydrostatic pressure promotes structural, functional, and metabolic maturation of hPSC-ECTs
• Endothelial cells are critical for maturation and vascular network formation
• Trained ECTs improve cardiac function and myocardial repair after infarction

## Graphical abstract

**Figure:**
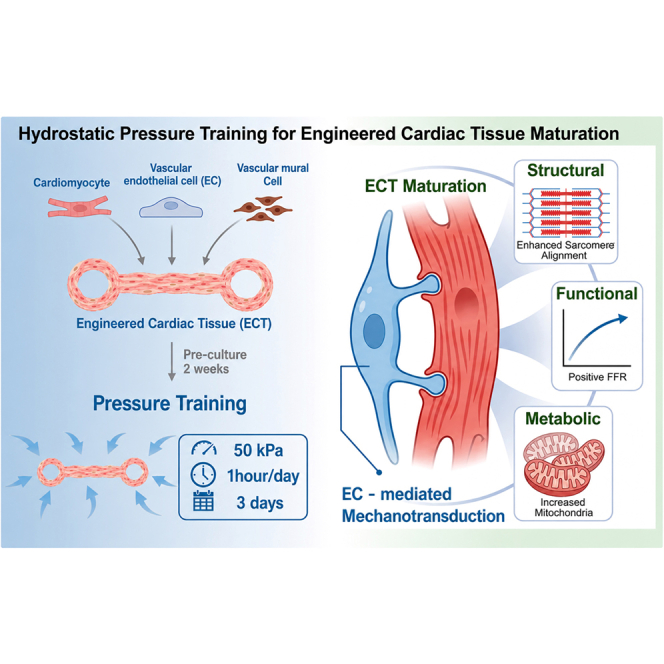

## Teaser

Masumoto et al. show that brief intermittent hydrostatic pressure training enhances maturation of human pluripotent stem cell-derived engineered cardiac tissues. This biomechanical stimulation improves tissue architecture, contractile performance, and mitochondrial function through endothelial cell-dependent mechanisms, supporting applications in disease modeling, drug screening, and regenerative medicine.

## Introduction

Cardiovascular diseases rank as the primary global cause of death (World Health Organization, 2021). The human heart possesses a limited ability to regenerate; when cardiomyocytes (CMs) are lost due to disease, they are replaced by non-contractile scar tissue, resulting in the thinning of the ventricular wall and impaired contractility. This sequence of events subsequently triggers remodeling, hypertrophy, and eventual heart failure (Pasumarthi and Field, 2002). Given the constraints of current treatments for damaged hearts, such as heart transplantation and artificial heart devices, there has been an increasing interest in the past few decades in utilizing CMs derived from human pluripotent stem cells (hPSCs), including embryonic stem cells (ESCs) and human-induced pluripotent stem cells (hiPSCs), as a promising avenue for cell-based cardiac regeneration therapy (Jebran et al., 2025; Shiba et al., 2012). Differentiated CMs from hPSCs (hPSC-CMs) share many characteristics with primary human CMs. Nonetheless, their effectiveness is hindered by their resemblance to immature human CMs at embryonic or fetal stages, leading to functional and structural differences from mature human CMs (Robertson et al., 2013). In response, numerous promising strategies have been explored and developed to generate mature hPSC-CMs such as extended culture periods (>100 days; Lundy et al., 2013) and biomechanical stimulation (Ronaldson-Bouchard et al., 2018; Ruan et al., 2016).

In an effort to replicate *in vivo* micro-environments, scientists have also advanced a three-dimensional (3D) culture system. This system involves co-culturing non-CMs and extracellular matrix (ECM) components, coupled with increased substrate stiffness (Nunes et al., 2013). Twenty years ago, the successful creation of cardiac tissue from embryonic chicken CMs marked the inception of 3D cardiac tissue engineering (Zimmermann et al., 2004). Numerous studies involving cardiac cell therapy utilizing 3D-engineered cardiac tissues (ECTs) created by hPSC-CMs have achieved success in their attempts to restore myocardial function in preclinical animal studies (Masumoto et al., 2016; Nakane et al., 2017). However, up to the present day, the therapeutic strategy has not been introduced into standardized clinical practice, nor have they seen widespread use in target validation and preclinical drug testing. The potential exists for a significant breakthrough if a mature ECT could be delivered to the damaged myocardium, effectively restoring ventricular function through synchronized beating with the heart, or disease modeling and drug discovery based on the mature ECTs highly recapitulating human heart tissue function.

Recent studies have highlighted that non-CM populations, including stromal cells and vascular endothelial cells (ECs), actively contribute to CM maturation rather than serving solely as structural support. For instance, Giacomelli et al. demonstrated that human iPSC-derived cardiac stromal cells enhance maturation and functional properties of engineered cardiac microtissues, underscoring the importance of multicellular interactions in cardiac tissue development (Giacomelli et al., 2020). Moreover, ECs are intrinsically exposed to mechanical stimuli such as shear stress and pressure and function as key mechanosensors within cardiovascular tissues. Through mechanotransduction pathways, ECs regulate ECM remodeling, paracrine signaling, and cell-cell interactions (Kutys and Chen, 2016), all of which may indirectly influence CM maturation and tissue-level functional organization. These observations provide a biological rationale for focusing on EC-mediated responses when investigating mechanically induced maturation of ECTs.

A previous study reported that the fluid pressure impacts the force of muscle contractions (Sleboda and Roberts, 2020). Additionally, alterations in ventricular pressure have been observed in mouse embryos during development. This revealed a notable increase in peak systolic pressure, rising from 2.13 ± 0.36 mmHg at embryonic day 9.5–11.15 ± 0.54 mmHg at embryonic day 14.5 (Ishiwata et al., 2003), indicating the involvement of pressure loading for normal development of the heart. Furthermore, a study on mice have shown that inducing temporary loading of high pressure for left ventricle through ascending aortic constriction and relief can stimulate cardiac repair associated with CM maturation and proliferation (Unno et al., 2019). Based on these findings, it would be possible that pressure load stimulation plays a significant role in the maturation of ECT. We also reported that positive pressure stimulates the progression of the cell cycle in ECs, thereby boosting their growth (Schwartz et al., 1999). There is an implication that these ECs incorporated within the ECT might respond to pressure load, potentially leading to maturation at both the cellular and tissue levels. In the present study, our hypothesis centered around the potential of hydrostatic pressure to enhance the maturation of hiPSC-derived ECTs (hiPSC-ECTs). Through our findings, we discovered that applying high and intermittent hydrostatic compression stimulation fosters the maturation of ECTs derived from hiPSCs that was mediated by ECs.

## Results

### Optimization of pressure training for ECTs

**Figure 1:**
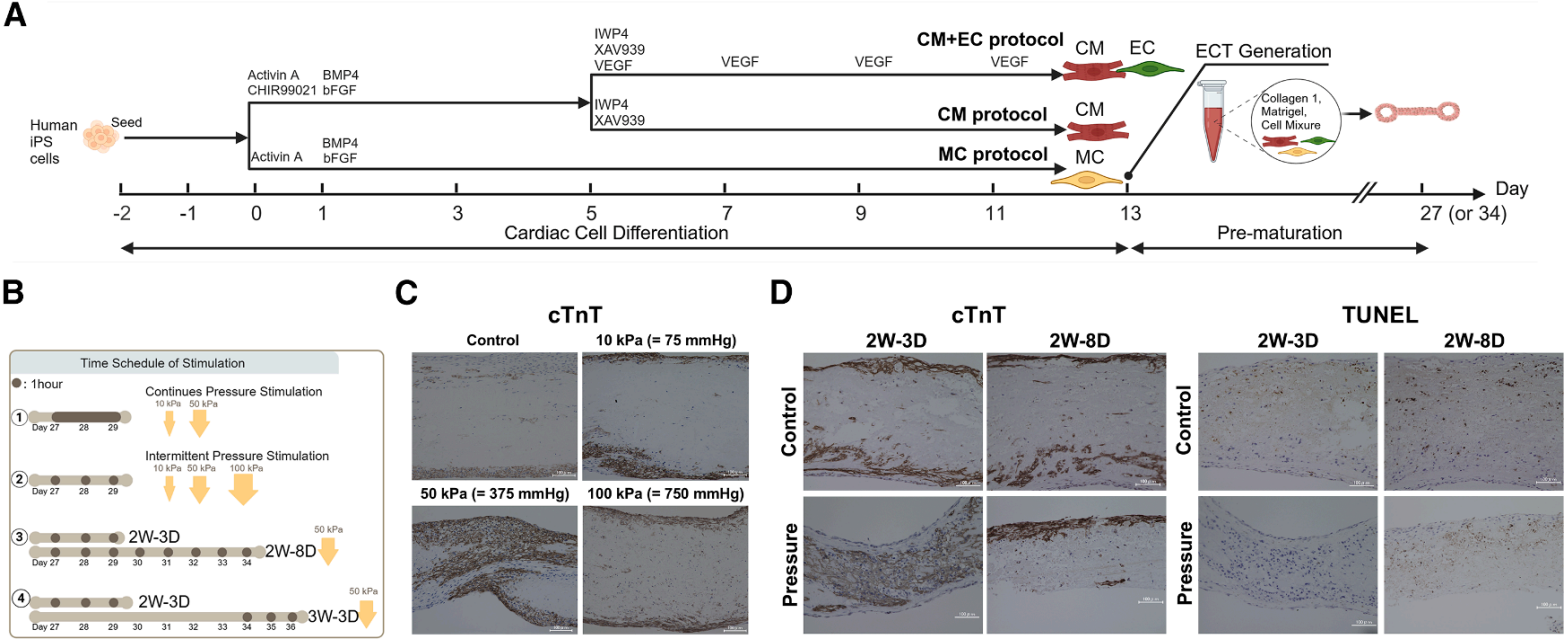
Optimizing hydrostatic pressure training for enhanced maturation of engineered cardiac tissues (A) Schematic diagram for cardiovascular cell induction protocols and generation of ECTs containing cardiomyocytes (CMs), endothelial cells (ECs), and mural cells (MCs). Cells and matrix were poured into custom PDMS molds on day 13 and then matured *in vitro*. (B) Hydrostatic pressure stimulation protocols. (C) Representative immunostaining of ECTs for cardiac isoform of troponin-T (cTnT) under different hydrostatic pressure in intermittent training. *n* = 3 independent ECTs derived from 3 independent differentiation experiments. Scale bars, 100 μm. (D) Representative immunostaining for cTnT (left) and terminal deoxynucleotidyl transferase dUTP nick end labeling (TUNEL) staining (right) of ECTs in different pressure stimulation duration. 2W-3D: pre-maturation 2 weeks and stimulation 3 days; 2W-8D: pre-maturation 2 weeks and stimulation 8 days. *n* = 7 independent ECTs derived from 7 independent differentiation experiments. Scale bars, 100 μm.

**Figure 2:**
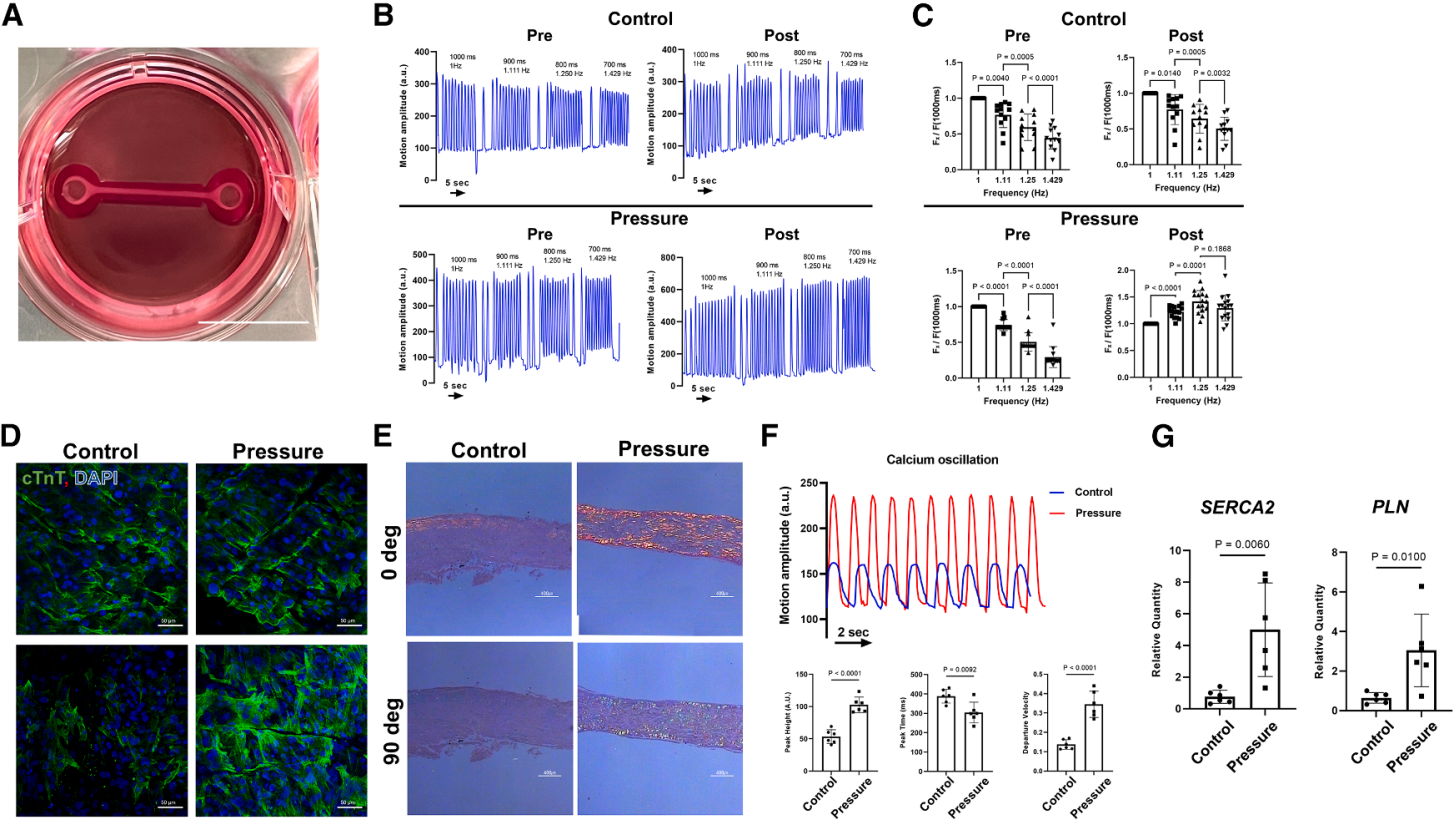
Enhanced functional and structural maturation of engineered cardiac tissues through optimized hydrostatic pressure training (A) Representative image of pressure-trained ECT after optimized protocol. Scale bars, 1 cm. (B and C) Representative waveform (Control [*n* = 12] and Pressure [*n* = 16] independent ECTs derived from 12 independent differentiation experiments) and (C) Quantification in the motion amplitude in accordance with the interval and frequency of external electrical stimulation (Control [*n* = 12] and Pressure [*n* = 16] independent ECTs derived from 12 independent differentiation experiments). Motion amplitude values were normalized to the baseline amplitude at 1,000 ms (1 Hz) for each ECT. All data are presented as relative values, with 1.0 corresponding to the amplitude at 1 Hz. An increase in normalized amplitude at higher stimulation frequencies indicates a positive force-frequency relationship (FFR). (D) Representative immunofluorescent staining of ECTs by confocal microscopy. DAPI: 4′,6-diamidino-2-phenylindole. *n* = 6 independent ECTs derived from independent differentiation experiments. Scale bars, 50 μm. (E) Polarized light microscopy for Sirius-red staining. Yellow area in 0° indicates higher collagen alignment along the direction of the pressure stimulation. *n* = 6 independent ECTs derived from independent differentiation experiments. (F) Representative calcium oscillation in spontaneous beating (upper) and peak height, peak time, and departure velocity (lower). *n* = 6 independent ECTs derived from independent differentiation experiments. (G) RT-qPCR. *SERCA2*: sarcoplasmic/endoplasmic reticulum Ca2+-ATPase, *PLN*, phospholamban. *n* = 6 independent ECTs derived from independent differentiation experiments. Data are presented as mean ± standard deviation (SD).

We generated hiPSC-ECTs using a previously established co-culture system consisting of hiPSC-derived cardiovascular cells and ECM proteins (Maihemuti et al., 2024; Figures 1A; S1, and S2). Initially, we sought to optimize how hydrostatic pressure should be applied to ECTs. Because ECTs only partially recapitulate native cardiac tissue, we hypothesized that continuous pressure loading, similar to that experienced by the heart *in vivo*, might induce tissue damage. To test this possibility, ECTs were subjected to continuous cyclic pressure stimulation at physiological (10 kPa, equivalent to 75 mmHg (Quantities, 1993) and non-physiological (50 kPa, equivalent to 375 mmHg) levels (Figure 1B) with the cyclic pressure loading pattern recapitulating normal systolic and diastolic phases of the heart beating (Figure S3). Histological analyses showed that continuous pressure loading did not increase the CM content of ECTs regardless of pressure magnitude (Figure S4). We therefore evaluated intermittent pressure loading at 10, 50, and 100 kPa for 1 h/day over 3 days (Figures 1B and 2). ECTs (CM: 39.12%; EC: 26.49%; MC: 12.81%) exposed to 50 kPa exhibited a higher proportion of cTnT-positive CMs (Figures 1C and S5A), increased live-cell density (Figure S5B), and elevated expression of *TNNT2*, encoding the cardiac isoform of troponin T, *TNNI3*, a marker of mature myofibers, and *CACNA1C*, encoding a subunit of the L-type calcium channel (Figure S5C). These findings indicate that intermittent hydrostatic pressure stimulation at 50 kPa promotes the structural maturation of ECTs.

**Figure 3:**
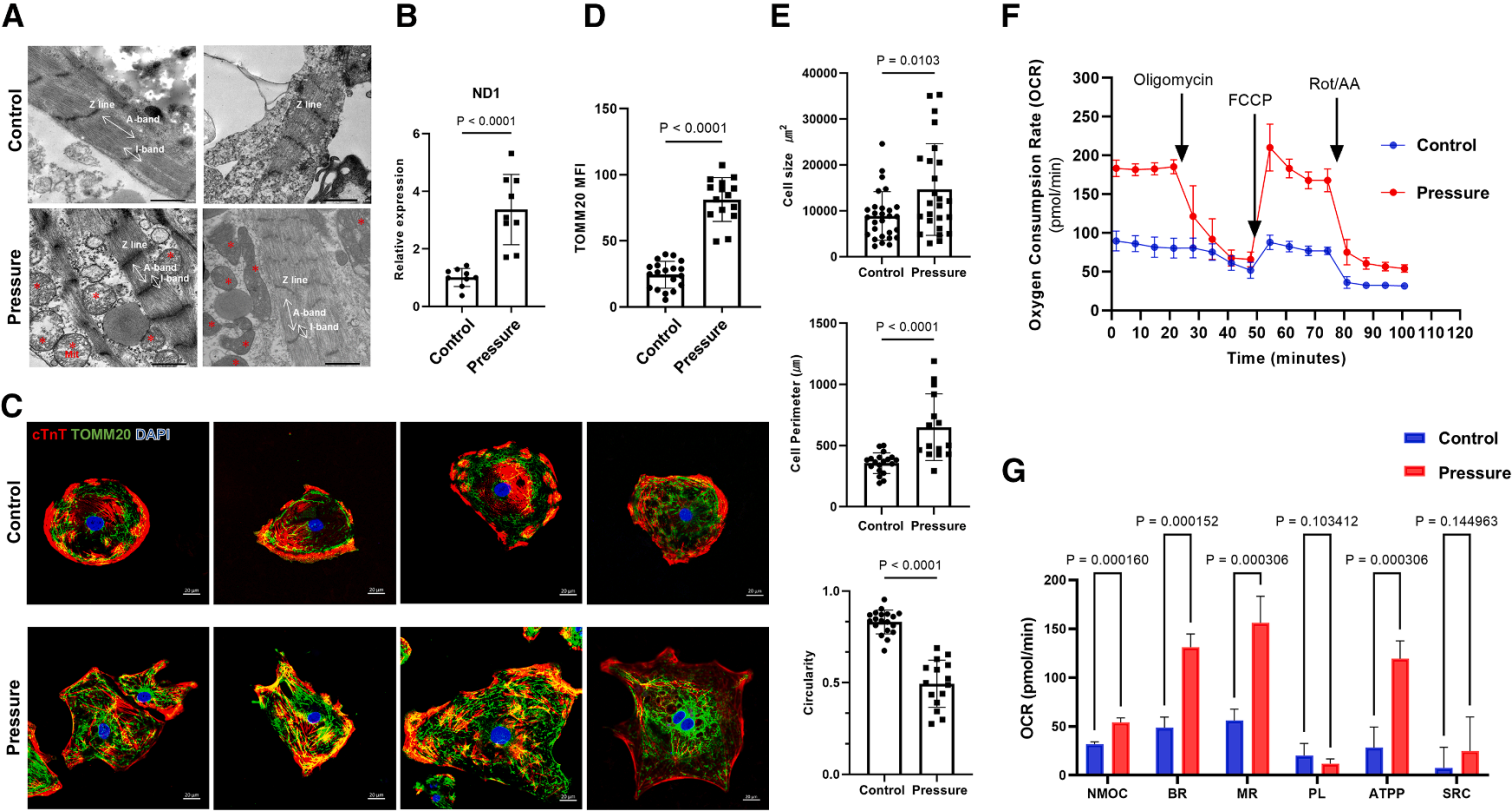
Enhanced metabolic maturation of engineered cardiac tissues (A) Representative transmission electron microscopic images. Z line, A-band, and I-band were indicated. Red asterisks indicate mitochondria (Mt). *n* = 3 independent ECTs derived from independent differentiation experiments. Scale bars, 1 μm. (B) RT-qPCR for NADH dehydrogenase 1 (ND1), indicating mitochondrial DNA. *n* = 9 independent ECTs derived from independent differentiation experiments. (C) Representative immunofluorescent staining of dissociated single cardiomyocytes. TOMM20 was used as a mitochondrial marker. *n* = 15 independent ECTs derived from 6 independent differentiation experiments. Scale bars, 20 μm. (D and E) Mean fluorescence intensity (*n* = 15 independent ECTs derived from 6 independent differentiation experiments) and (E) cell size (Control [*n* = 28], Pressure [*n* = 25] ECTs derived from 6 independent differentiation experiments), cell perimeter (Control [*n* = 19], Pressure [*n* = 15] ECTs derived from 6 independent differentiation experiments), and circularity (Control [*n* = 19], Pressure [*n* = 15] ECTs derived from 6 independent differentiation experiments) in accordance with immunofluorescent staining of dissociated single cardiomyocytes. (F and G) Oxygen consumption rate (OCR). NMOC, non-mitochondrial oxygen consumption; BR, basal respiration; MR, maximal respiration; PL, proton leak; ATPP, ATP production; SRC, spare respiratory capacity. *n* = 3 independent ECTs derived from independent differentiation experiments. Data are presented as mean ± standard deviation (SD).

Next, we determined the optimal duration of intermittent pressure stimulation by comparing 3 days (1 h/day; 2W-3D) and 8 days (1 h/day; 2W-8D) following 2 weeks of pre-maturation culture (Figures 1B, 2, and 3). Histological analyses revealed that 2W-3D significantly increased myocardial density and myocardial tissue occupancy compared with 2W-8D (Figures 1D, S6A). In addition, CMs in 2W-3D were densely concentrated in the central region of the ECT, exhibiting a structure resembling mature rat papillary muscle (Figures S6B and S6C), whereas these features were absent in 2W-8D. The 2W-3D group also showed greater collagen alignment (Figure S6D) and lower apoptosis (Figure 1D), suggesting that prolonged stimulation was detrimental to tissue integrity. Contractile function was evaluated using MUSCLEMOTION, a versatile video-based system that quantifies the motion amplitude of the ECTs (Sala et al., 2018) (Figure S6E). Although motion amplitudes decreased with increasing pacing frequency in 2W-8D, they increased in 2W-3D as stimulation frequency was raised from 1,000 ms (1 Hz) to 800 ms (1.25 Hz), indicating the presence of a positive force-frequency relationship (FFR), a hallmark of mature cardiac tissue (Endoh, 2004). Consistent with these findings, quantitative real-time PCR (qRT-PCR) analyses demonstrated upregulation of *TNNT2*, *TNNI3*, and *CACNA1C* in 2W-3D but not in 2W-8D (Figure S6F). Together, these results indicate that intermittent stimulation for 3 days (1 h/day; total 3 h) is sufficient to promote ECT maturation, whereas longer stimulation is disadvantageous.

**Figure 4:**
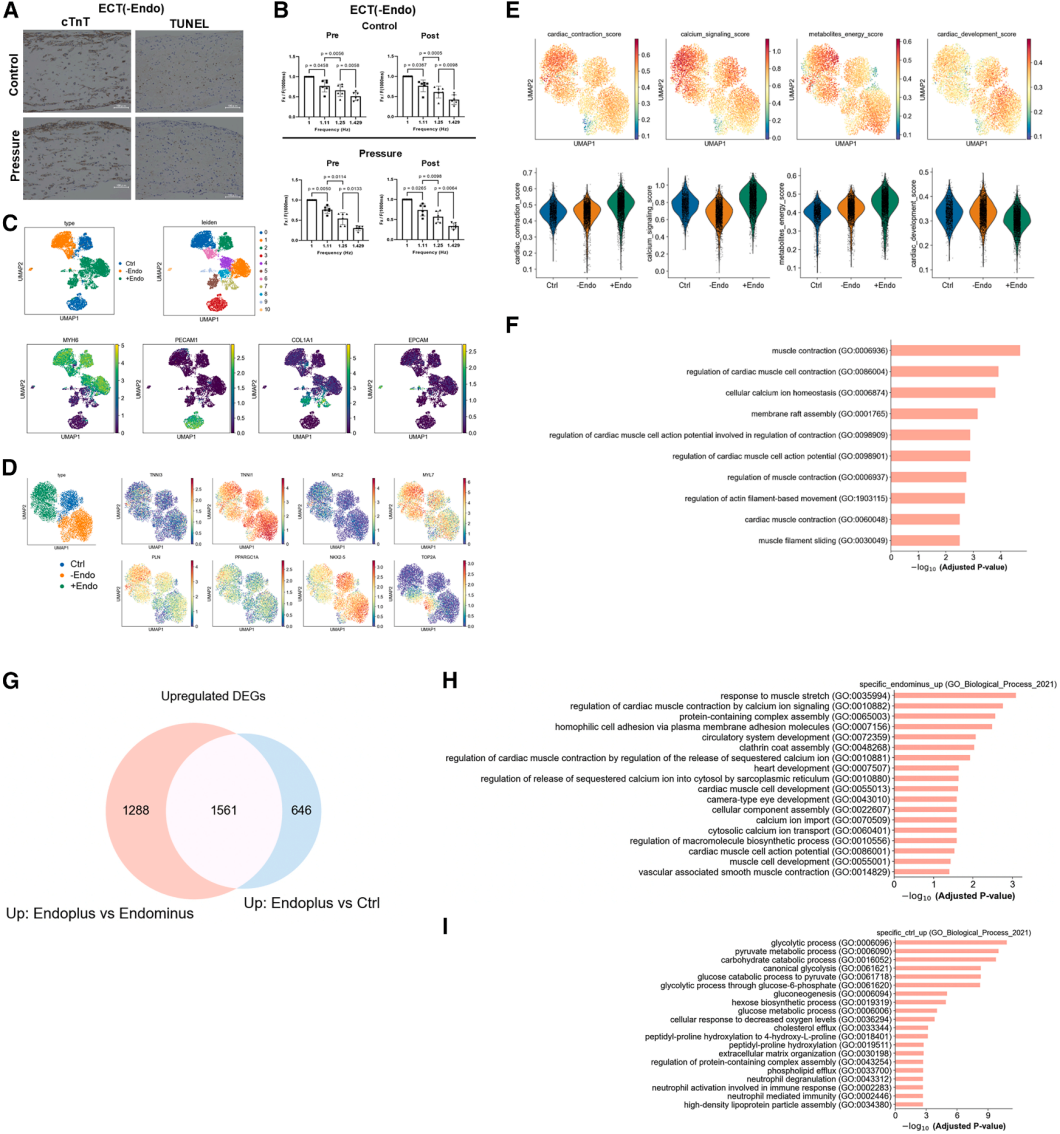
Maturation of engineered heart tissues was mediated by vascular endothelial cells (A) Representative immunostaining for cTnT (left) and TUNEL staining of ECTs (right) of ECTs without vascular endothelial cells (ECT[−Endo]). *n* = 6 independent ECTs derived from independent differentiation experiments. Scale bars, 100 μm. (B) Quantified changes in the motion amplitude in accordance with the frequency of electrical stimulation in ECT(−Endo). *n* = 6 independent ECTs derived from independent differentiation experiments. Notably, ECT(−Endo) did not exhibit a positive FFR after pressure training, indicating a lack of functional maturation in the absence of endothelial cells. This image is intended to highlight the limitation of ECTs without ECs and to emphasize the requirement of endothelial cells for pressure-induced cardiomyocyte maturation. (C–F) Single-cell RNA-seq. (C) Analyses for total population. Uniform manifold approximation and projection (UMAP) by samples (left) and clusters (right) are shown in the upper part. Feature plot analyses using marker genes for each cell type are shown in the lower part. (D–F) Analyses for CM subpopulation. (D) Feature plot for maturation-related markers. (E) Feature and violin plot for gene scoring using the gene sets indicative of specific cell functions. (F) Gene Ontology (GO) analysis for differentially expressed genes (DEGs) in ECT(+Endo) sample. Differential expression analysis was performed by comparing the Endo+ group against the remaining groups combined. Data are presented as mean ± standard deviation (SD). (G) Venn diagram illustrating the number of upregulated DEGs in the pressure-treated ECT(+Endo) (Endoplus) group compared with the pressure-treated ECT(−Endo) (Endominus) group and the control (Ctrl) group. The overlapping region shows 1,561 genes that are commonly upregulated in both comparisons. (H) Gene ontology (GO) analysis (Biological Process, 2021) for genes specifically upregulated in the Endoplus group relative to the Endominus group (specific_endominus_up). (I) GO analysis (Biological Process, 2021) for genes specifically upregulated in the Endoplus group relative to the Ctrl group (specific_ctrl_up). For (B) and (C), the *x* axis represents –log10 (adjusted *p* value). For (C) to (I), *n* = 1 ECT per condition derived from three independent differentiation experiments.

As reported previously, early CMs are more responsive to physical stimulation than later-stage CMs (Ronaldson-Bouchard et al., 2018), suggesting that the timing of training may influence tissue maturation. We therefore compared pressure training initiated after 2 weeks (2W-3D) or 3 weeks (3W-3D) of pre-maturation culture (Figures 1B, 2, 3, and 4). ECTs cultured for only 1 week were not synchronized (Video S1), indicating insufficient maturation, and were therefore excluded from further analyses. Histological analyses showed that both training protocols increased the cTnT-positive ratio and CM volume compared with their respective controls (Figures 1D and S7A–S7C), with more pronounced effects in the 2W-3D group. Although pressure-trained tissues tended to be slightly thinner than controls, this difference was not statistically significant (Figure S7D). Positive FFRs were observed in both groups (Figure S7E), and expression of *TNNT2*, *TNNI3*, and *CACNA1C* was increased under both conditions (Figure S7F). Overall, pressure training promoted ECT maturation in both groups, but the effect was greater when initiated after 2 weeks of pre-maturation culture. Based on these findings, we established the final protocol as 50 kPa for 1 h/day over 3 days following 2 weeks of pre-maturation culture (Figure 2A), which was used in all subsequent analyses.

Video S1. Microscopic observation of ECT at day 7 after formation

### Pressure training enhances ECT contractility

Because positive FFR was not consistently observed in all experiments, we investigated whether the CM composition of ECTs was associated with pressure responsiveness. We found that the CM proportion was significantly higher in ECTs exhibiting positive FFR (63.5% ± 6.3%) than in those with negative FFR (45.9% ± 4.0%) (Figure S8A). Pressure training increased the expression of *TNNT2*, *TNNI3*, *CACNA1C*, and *TNNI3/TNNI1* only in the positive FFR group (Figure S8B), whereas no significant changes were observed in the negative FFR group (Figure S8C). These findings suggest that a CM composition ratio of 57.2%–69.8% (95% confidence interval [CI]) is favorable for generating ECTs responsive to pressure training.

Next, we evaluated the effect of the optimized training regimen on functional maturation by assessing FFR before and after training (Figures 2B, 2C, and S9). In the control group, motion amplitudes decreased with increasing pacing frequency both before and after training, showing a strong negative correlation (Video S2). In contrast, pressure-trained ECTs exhibited increased motion amplitudes with increasing stimulation frequency after training, despite showing a negative response before training, indicating functional maturation (Video S3). However, the peak FFR was observed at approximately 1.25 Hz, which is lower than the reported peak frequency of 2.0–2.5 Hz in human myocardium (Endoh, 2004).This finding suggests that pressure training alone may not be sufficient to achieve full adult-like maturation, potentially due to limitations in tissue formation conditions, including ECM composition. Pressure-trained ECTs also exhibited greater CM alignment than controls, which may contribute to the enhanced contractile performance observed after training (Figure 2D). In addition, polarization microscopy demonstrated improved collagen alignment in pressure-trained ECTs compared with controls (Figure 2E).

Video S2. FFR of ECT without pressure training (control) The frequency of external electrical stimulation increased from 1,000 to 700 ms

Video S3. FFR of ECT after pressure training The frequency of external electrical stimulation increased from 1,000 to 700 ms

We hypothesized that enhanced tissue contractility following pressure training was mediated, at least in part, by improved calcium handling, as suggested by qRT-PCR analyses (Figures 1G and S7E). To test this, intracellular calcium transients were visualized using a calcium indicator. Pressure-trained ECTs exhibited significantly increased peak calcium flux and faster calcium uptake and release during spontaneous beating (Figure 2F; Video S4) and electrical stimulation (Figure S10), indicating enhanced calcium handling capacity. Consistent with these findings, pressure training significantly increased the expression of the sarcoplasmic reticulum (SR)-associated proteins *SERCA2* and *PLN* (Figure 2G), suggesting improved SR function.

Video S4. Calcium oscillation of ECT(+Endo) MCTR; mechano-culture training (pressure training)

### Pressure training promotes metabolic function

As an additional mechanism contributing to tissue maturation, we conjectured that pressure training might enhance the metabolic maturation of the ECTs (Mills et al., 2017). This notion was supported by our observations through transmission electron microscopy, which revealed a substantial increase in the mitochondrial fraction located between sarcomeric fibers in the pressure-treated samples (Figure 3A). Notably, there was also a significant enrichment of mitochondrial DNA in the pressure-treated samples (Figure 3B). To assess both the mitochondrial components and the morphological characteristics of the CMs within the ECTs, we isolated CMs from the ECTs using enzymatic dissociation. The debris removal process proved successful in yielding a higher percentage of live cells in both control and pressure-treated samples (Figure S11). Using confocal microscopy, we evaluated the TOMM20-positive mitochondrial components and confirmed that the positive TOMM20 area was larger in isolated CMs from pressure-trained ECTs compared with those in control ECTs (Figures 3C and 3D). Moreover, CMs isolated from pressure-treated ECTs exhibited a significantly larger size and reduced circularity, indicating maturation at the cellular level (Figures 3C and 3E). Employing a flux analyzer to assess metabolic function, we noted a significant increase in the maximal oxygen consumption rate (OCR) and ATP production in pressure-treated ECTs as compared with the control group, indicating metabolic maturation of pressure-trained ECTs (Figures 3F and 3G).

### ECs are essential for the hiPSC-ECT maturation

Next, we investigated how ECs work in response to hydrostatic pressure stimulation toward tissue maturation. We generated ECTs with EC [ECT(+Endo)] and without EC [ECT(−Endo)], respectively (Figure S12A). We found that ECT(+Endo) started to shrink just after ECT formation, whereas no apparent tissue shrinkage was observed in ECT(−Endo), indicating higher ECM digestion capacity in ECT(+Endo) compared with that in ECT(−Endo) immediately after ECT formation (Figure S12B). The timing of tissue shrinkage due to gel compaction (Figure S12C), and the timing of the initiation of tissue beating (Figure S12D) was earlier in ECT(+Endo) than in ECT(−Endo), indicating more rapid tissue maturation in ECTs with ECs. We also found that the structural maturation evaluated by histological studies (Figures 4A and S12E), functional maturation validated by FFR (Figure 4B), and enhanced calcium turnover capacity (Figure S12F; Video S5) were canceled in ECT(−Endo) after pressure training. Importantly, Figure 4B specifically illustrates that ECTs lacking ECs fail to exhibit a positive FFR under the optimized pressure-training protocol, thereby highlighting the requirement of ECs for the acquisition of frequency-dependent contractile function.

Video S5. Calcium oscillation of ECT(-Endo) MCTR; mechano-culture training (pressure training)

To further evaluate tissue and CM maturation facilitated by the pressure training, we conducted single-cell RNA-seq analysis for samples: no training for ECT(+Endo) (Ctrl), pressure training for ECT(−Endo), and pressure training for ECT(+Endo). We identified 11 distinct clusters in the total population, annotated as CM (clusters 0, 1, 2, 4, and 6), EC (cluster 3), fibroblast-like stromal cells (clusters 5 and 7), epithelial cells (clusters 9 and 10), and stress-responsive fibroblasts (cluster 8) (Figures 4C and S13A). Next, we conducted a subpopulation analysis of CM (clusters 0, 1, 2, 4, and 6). CMs were clearly identified as distinct clusters between the samples (Figure 4D). The expression of the gene marker indicative of cardiac maturation was significantly different across the samples, demonstrating an increase in sarcomere constituent markers associated with maturation (*TTNI3*, *MYL2*, *MYL7*, and *MYH7*), and the SR constituent marker *PLN* were highly expressed in ECT(+Endo). Additionally, metabolic-related markers such as *PPARGC1A* were also highly expressed in ECT(+Endo). Conversely, markers indicating early CM development, such as *NKX2-5*, were expressed at lower levels compared with other samples. No differences in the expression of proliferation markers like *TOP2A* were observed between samples (Figures 4D, S13B, and S13C). Furthermore, we performed gene scoring analysis using the gene sets indicative of specific cell functions. The results showed that the scores for genes related to CM functions such as cardiac contraction, calcium signaling, and metabolic energy were significantly higher in ECT(+Endo) compared with other samples. In contrast, genes associated with early cardiac development were downregulated in ECT(+Endo) (Figure 4E). Gene ontology (GO) analysis for differentially expressed genes (DEGs) in ECT(+Endo) sample listed multiple GO terms related to cardiac muscle contraction (Figure 4F). These findings indicate that hydrostatic pressure training promotes maturation and function at the cellular level of the constituent CMs. Moreover, the presence of ECs is necessary for this maturation process.

To further dissect the synergistic effects of ECs and hydrostatic pressure, we categorized upregulated DEGs across three groups, non-trained ECT(+Endo) (Ctrl), pressure-trained ECT(−Endo) (Endominus), and pressure-trained ECT(+Endo) (Endoplus) using a Venn diagram. This experimental design allows us to discern the specific contribution of ECs to the mechanical response by using the pressure-trained ECT(−Endo) group as a reference for the direct effects of pressure in the absence of endothelial interactions. Venn analysis identified 1,561 shared DEGs that were significantly upregulated in Endoplus relative to both Ctrl and Endominus (Figure 4G). Gene Ontology (GO) enrichment analysis of these shared genes demonstrated that EC-dependent upregulation (Endoplus vs. Endominus) was predominantly associated with structural reorganization processes, including protein-containing complex assembly and homophilic cell adhesion (Figure 4H), indicating a critical role for ECs in coordinating tissue-level maturation in response to mechanical stimuli. Furthermore, comparison of Endoplus with Ctrl revealed a distinct metabolic shift in EC-containing tissues under pressure, characterized by enrichment of glycolytic process, pyruvate metabolic process, and cellular response to decreased oxygen levels (Figure 4I). Collectively, these findings demonstrate that EC-mediated microenvironmental regulation and hydrostatic pressure act synergistically to drive ECT maturation, integrating metabolic remodeling, structural organization, and functional adaptation.

### Mature ECTs promote myocardial regeneration

**Figure 5:**
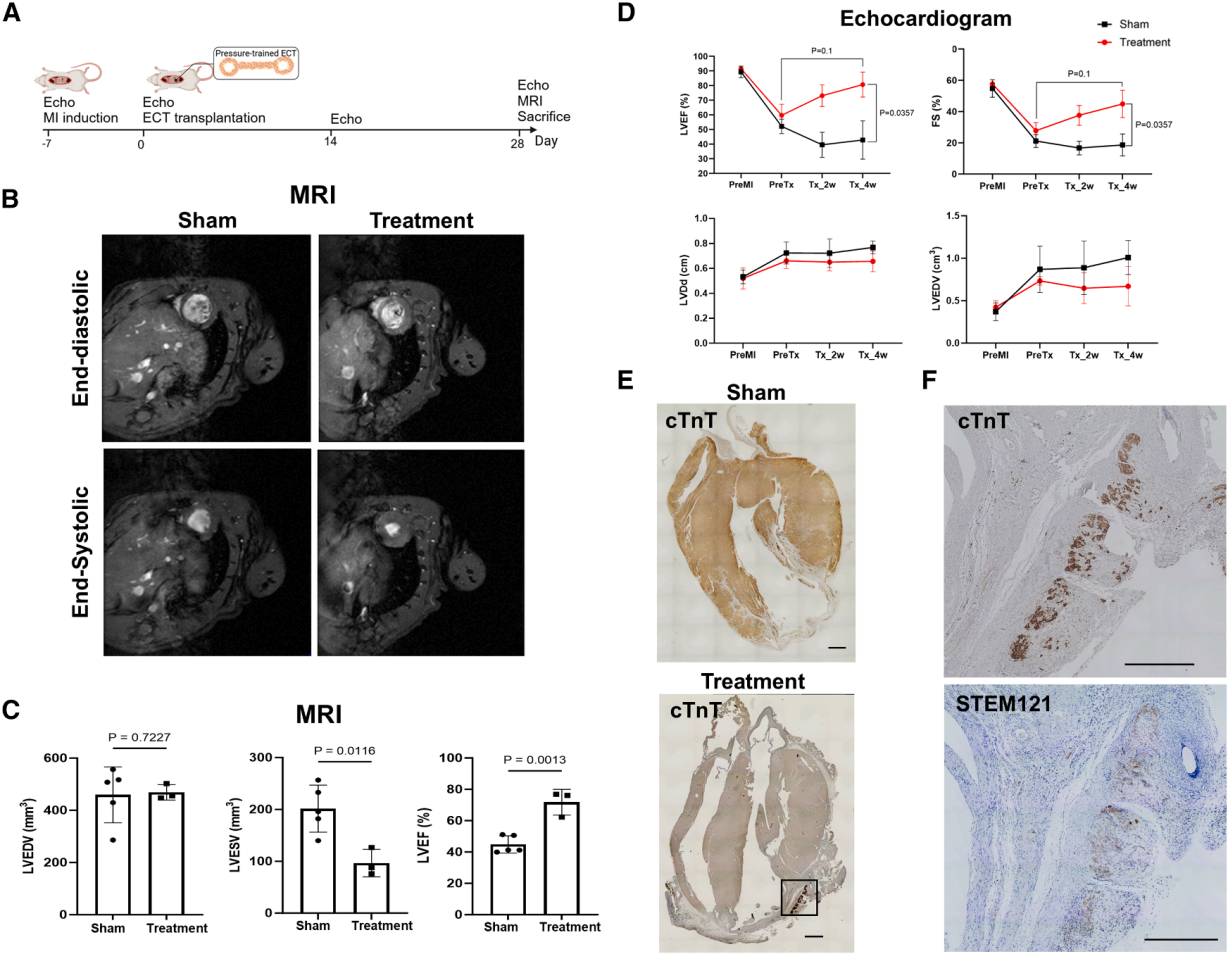
Cardiac functional recovery and myocardial regeneration through transplantation of pressure-trained engineered cardiac tissues (A) Schematic timeline of *in vivo* experiment. MI, myocardial infarction; Echo, echocardiogram; MRI, magnetic resonance imaging. (B) Representative MRI images of base cross-sections of the rat hearts at 4 weeks after pressure-trained ECT transplantation at end-diastolic and end-systolic phases. (C) Left ventricular end-diastolic volume (LVEDV), left-ventricular end-systolic volume (LVESV), and left ventricular ejection fraction (LVEF) evaluated by MRI (*N* = 5 [sham] and 3 [Treatment]). Sham (*n* = 5) and Treatment (*n* = 3) ECTs derived from 3 independent differentiation experiments. (D) Echocardiogram (*N* = 5 [sham] and 3 [Treatment]). Sham (*n* = 5) and Treatment (*n* = 3) ECTs derived from 3 independent differentiation experiments. Left ventricular ejection fraction (left upper), Fractional shorting (right upper), left ventricular end-diastolic diameter (left lower), and left ventricular end-diastolic volume (right lower). Pre MI: before induction of MI; PreTx: before treatment; Tx_2w/4w, 2/4 weeks after treatment. (E) Representative cTnT immunostaining of heart sections (long axis). Scale bars, 1,000 μm. (F) Higher magnification images of engrafted ECTs (indicated in E). cTnT (upper) and STEM121, a human cytoplasm marker (lower). Scale bars, 1,000 μm. Data are presented as mean ± standard deviation (SD).

Next, we investigated the effects of the transplantation of the pressure-trained mature ECT onto an animal disease model in cardiac functional recovery and myocardial regeneration. Following the induction of myocardial infarction through permanent ligation of the left anterior descending coronary artery in rats, we reopened the chest 1 week later and transplanted the trained ECTs to the infarcted area (Figure 5A; Video S6). One month after the transplantation, cardiac MRI showed no significant difference in end-diastolic volume of the left ventricle compared with the sham control group. However, the end-systolic volume significantly decreased in the treatment group, resulting in a significantly higher ejection fraction in the treatment group (Figures 5B and 5C). Subsequently, we evaluated the size and contractile function of the hearts in both the sham control group and the treatment group after ECT transplantation in a double-blind manner using echocardiographic examinations to track the temporal changes in cardiac functional recovery. As depicted in Figure 5D, the transplantation group exhibited a significant contrast with the sham control group as it curtailed the post-transplantation increase of the left ventricular end-diastolic volume. Additionally, both the ejection fraction and fractional shortening were significantly improved in the treatment group compared with the sham group up to 4 weeks post-transplantation.

Video S6. Transplantation of pressure-trained ECTs onto a rat myocardial infarction model

We examined whether human myocardial layers could regenerate within these rat myocardial infarction hearts (Figures 5E and 5F). In the treatment group, regenerated myocardial layer (cardiac troponin-T-positive) was observed along the ECT transplantation site. STEM121 staining, a marker for human cells, indicated that these regenerated myocardial layers on the heart surface were of human origin.

We further examined whether pressure-trained ECTs exert greater therapeutic effects compared with non-pressure-trained counterparts. Both types were transplanted into the same rat MI model, and cardiac function was assessed. At 2 weeks post-transplantation, pressure-trained ECTs showed higher tendency of therapeutic effects in both cardiac chocardiography (Figure S14). However, by 4 weeks post-transplantation, no difference in therapeutic efficacy was observed between the two groups.

### Disease modeling with patient-derived ECTs

Next, we explored whether this pressure training could promote maturation in ECTs carrying genetic abnormalities associated with heart disease. We applied the same training protocol as we did for healthy ECTs to the ECTs engineered using iPSCs established from a patient with familial-dilated cardiomyopathy (fDCM) (HPS1799; supplied by RIKEN BioResource Research Center, Tsukuba, Japan). In histological examination, we confirmed that, unlike what was observed in ECTs derived from healthy iPSCs, the structural maturation was not evident in ECTs derived from fDCM cells with this training (Figure S15A). Moreover, in the assessment of pulsatile function using MUSCLEMOTION, there was no shift toward a positive FFR as a result of the training (Figures S15B and S15C). Additionally, in the evaluation of calcium oscillations, the training did not lead to an improvement in peak height (Figures S15D and S15E; Video S7). These results suggest that ECTs derived from fDCM patient’s iPSCs exhibit limited maturation-promoting effects in response to pressure training, possibly replicating a pathological phenotype.

Video S7. Calcium oscillation of ECT derived from fDCM-patient iPSCs HPS1799; familial dilated cardiomyopathy patient-derived iPSC line, MCTR; mechano-culture training (pressure training)

### Vascular network promotes ECT function

**Figure 6:**
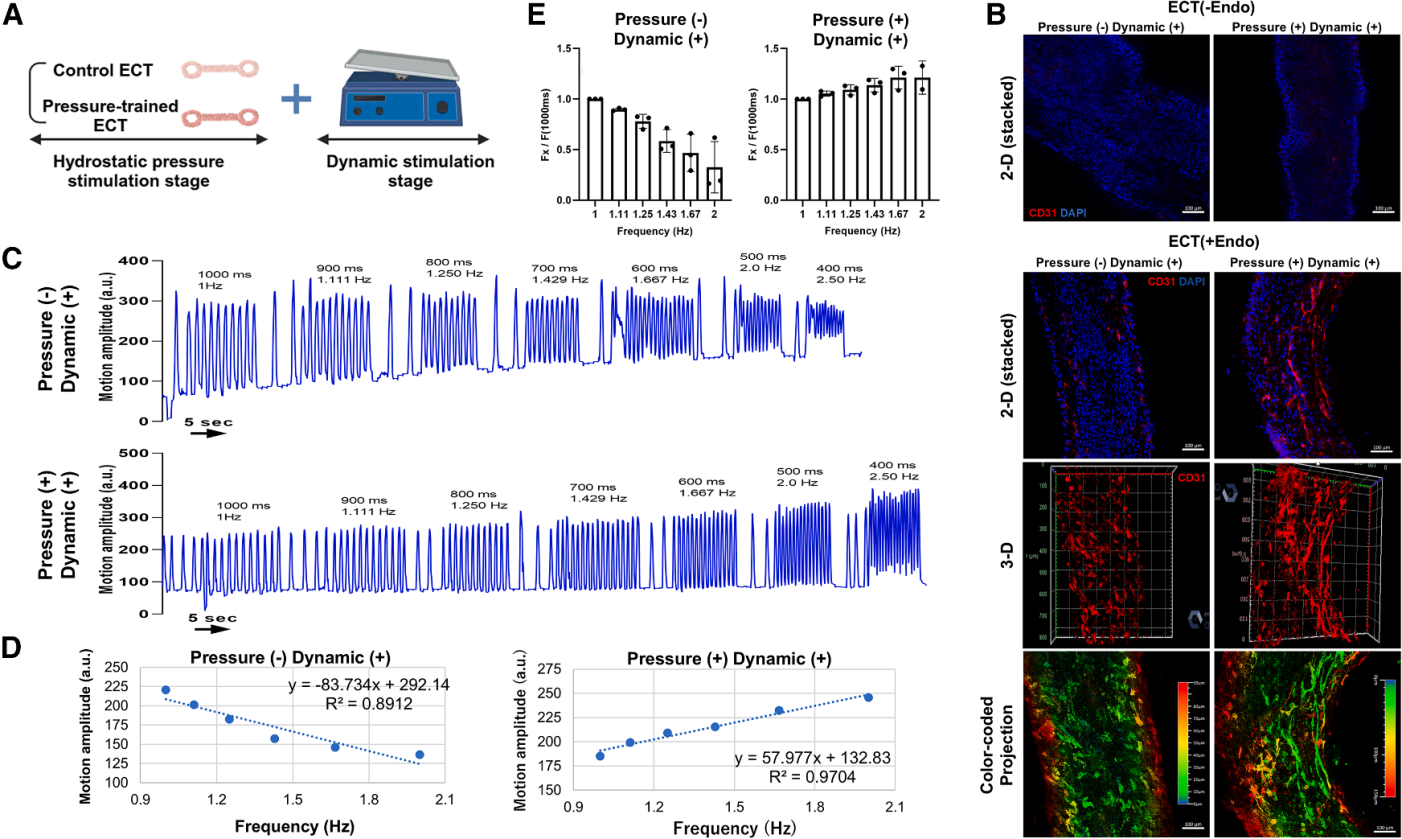
Combination of hydrostatic pressure and dynamic culture promotes vascular network formation and further enhances engineered cardiac tissue function (A) Schematic diagram of the combination of hydrostatic pressure and dynamic culture. (B) Representative CD31 confocal immunofluorescent imaging of the ECT(−Endo) (upper) and ECT(+Endo) (lower) after dynamic culture. *n* = 6 independent ECTs derived from independent differentiation experiments. Scale bars, 100 μm. (C) Representative waveforms in the motion amplitude in accordance with the interval and frequency of external electrical stimulation after dynamic culture. (D) Relationship between the frequency of electrical stimulation frequency (X) and the motion amplitude (Y) in the whole beating cycle. (E) Quantified changes in the motion amplitude in accordance with frequency of external electrical stimulation after dynamic culture. Data are presented as mean ± standard deviation (SD). For (C)–(E), *n* = 3 independent ECTs derived from independent differentiation experiments.

Vascular network formation is reported to be one of the fundamental factors for the establishment of mature heart organoid derived from hPSCs (Voges et al., 2023). Furthermore, it is reported that endothelial tube formation can be enhanced under hydrostatic pressure (Yoshino et al., 2020). By confocal microscopic imaging for ECTs, we tried to visualize the distribution of ECs among the ECT(+Endo) just after pressure training. Even though we could observe CD31-positive ECs in pressure-trained ECT(+Endo), we could not observe vascular network formation (Figure S16A). Previous studies have reported that dynamic culture using fluidic system promotes maturation of cardiac tissue derived from hPSCs (Gao et al., 2018; Jackman et al., 2016), and it also enhances vascular network formation in hiPSC-derived cardiac tissue containing vascular cells (Abulaiti et al., 2020; Arslan et al., 2023; Murata et al., 2024). Building on these findings, we extended dynamic culture for an additional week with these pressure-trained ECTs (Figure 6A). As a result, we confirmed the formation of a distinct vascular network within these ECT(+Endo), whereas the vasculature was absent in ECT(−Endo) even after the combination of pressure training and dynamic culture (Figures 6B and S16B; Videos S8 and S9). While vascular ECs were present in the ECT(+Endo) with dynamic culture alone, the formation of a vascular network was not observed unless pressure training was applied. By using color-coded projection imaging, we discovered that the vascular network developed at the center of the ECT(+Endo) where the myocardial layer formed, mirroring the process of vascular network formation in native heart tissue. The pressure-trained ECT(+Endo) with dynamic culture exhibited positive FFR up to the beating frequency of 2.0 Hz (120 bpm), which was close to that of adult human heart tissue (Endoh, 2004) (Figures 6C–6E; Videos S10 and S11).

Video S8. 3D-constructed confocal microscopical image of ECT [Pressure (-), Dynamic (+)]

Video S9. 3D-constructed confocal microscopical image of ECT [Pressure (+), Dynamic (+)]

Video S10. FFR of ECT [Pressure (-), Dynamic (+)]

Video S11. FFR of ECT [Pressure (+), Dynamic (+)]

## Discussion

In the present study, we identified a method to promote the maturation of hiPSC-derived CMs and ECTs using high intermittent hydrostatic pressure, requiring only 3 h of training over 3 days. Unlike stretch-based conditioning systems, this approach modifies the pressure environment without inducing direct tissue elongation. Various strategies have been developed to promote hPSC-CM maturation (Mills et al., 2017; Ronaldson-Bouchard et al., 2018; Tulloch et al., 2011; Yang et al., 2019). Because the heart is continuously exposed to cyclic pressure changes during both development and adult function, pressure loading may represent a physiologically relevant maturation cue. However, its effect on hPSC-derived cardiac tissues has not previously been demonstrated experimentally. Here, we show for the first time that pressure training promotes both structural and functional maturation of hPSC-derived CMs and ECTs. Importantly, we found that brief, high-intensity intermittent stimulation was sufficient to achieve these effects without apparent tissue damage. We selected 50 kPa as an exploratory stimulus intensity to evaluate the maturation-promoting potential of hydrostatic pressure in ECTs. Although this pressure exceeds physiological ventricular levels, previous mechanobiology studies have shown that hydrostatic pressures in the tens of kilopascals effectively activate mechanotransduction pathways in vascular cells, leading to alterations in gene expression, cytoskeletal organization, and cell function (Acevedo et al., 1993; Erkens et al., 2017; Sumpio et al., 1994; Thoumine et al., 1995). Furthermore, prolonged pressure training (8 days) increased apoptosis, suggesting that excessive mechanical stimulation may induce cumulative cellular stress and compromise cell survival. Consistent with previous reports in mechanically sensitive tissues (D'Lima et al., 2001; Hwang and Kim, 2015; Lee et al., 2005; Powers et al., 2005), our findings indicate that the short and intermittent protocol used here may provide an optimal balance between maturation-promoting stimuli and mechanical overexposure. The timing of mechanical stimulation is likely a critical determinant of its biological effect. Although CMs began spontaneous beating at an early stage, synchronized contractions across the entire ECT were not observed until day 27, when electromechanical coupling and tissue-level organization had been established. These findings suggest that pressure stimulation becomes effective only after a certain degree of maturation has been achieved, whereas earlier stimulation may primarily function as a non-specific stressor. Thus, a stage-appropriate application of pressure is likely essential for coordinated mechanotransduction and functional maturation.

In this study, we identified ECs as critical mediators of hydrostatic pressure-induced CM maturation. Previous studies have shown that ECs respond to hydrostatic pressure through mechanosensitive signaling pathways, resulting in substantial changes in cellular behavior (Acevedo et al., 1993; Erkens et al., 2017; Sumpio et al., 1994; Tamargo et al., 2023; Thoumine et al., 1995). Consistent with these observations, we found that ECs were essential for pressure-induced tissue maturation and that the combination of pressure training and dynamic culture promoted vascular network formation, which was associated with improved tissue function. The enhanced maturation observed in EC-containing ECTs may result from changes in the extracellular environment and increased CM-CM interactions (Figure S12), as previously suggested (Rodriguez et al., 2019). Together with accumulating evidence that non-CM populations support CM maturation through structural, paracrine, and cell-cell interactions (Giacomelli et al., 2020), our findings suggest that EC-mediated mechanotransduction represents an important pathway regulating maturation of ECTs under hydrostatic pressure.

The relationship between tissue alignment, contractile function, and molecular maturation warrants further investigation. In native myocardium, positive FFR arises from coordinated structural and functional properties, including CM alignment, sarcomere organization, SR function, and ECM architecture (Endoh, 2004; Godier-Furnemont et al., 2015). Importantly, these elements are functionally integrated at the tissue level rather than acting independently. Consistent with this concept, pressure-trained ECTs exhibited enhanced *TNNT2* expression, CM alignment, sarcomere organization, and calcium handling, suggesting that the observed positive FFR reflects an integrated maturation process. Previous studies have shown that anisotropic CM organization improves force transmission efficiency and contractile performance (Bian et al., 2014; Godier-Furnemont et al., 2015; Zimmermann et al., 2002), supporting the interpretation that improved alignment contributed to the functional benefits observed in our study. Nevertheless, a direct mechanistic relationship between alignment, contractile function, and gene expression remains to be established. Although glycolytic and hypoxia-responsive pathways were enriched, we interpret these changes as adaptive metabolic remodeling that supports tissue function under mechanically demanding conditions. On the other hand, due to technical limitations, we were unable to demonstrate direct vascular integration between the ECT-derived vascular network and host circulation after transplantation. Confirmation of functional vascular integration will be important in future studies to further establish the therapeutic potential of this strategy.

The functional and structural recovery observed following transplantation of pressure-trained ECTs highlights the potential of this strategy for cardiac regenerative medicine. Previous studies have suggested that transplantation of more mature hPSC-derived CMs may improve engraftment and myocardial regeneration while reducing arrhythmogenic risk (Kobayashi et al., 2024). However, arrhythmias were not a primary endpoint of this study, and their evaluation was limited by the use of rat models with substantially higher heart rates than humans. Future studies using large-animal models will therefore be necessary to assess arrhythmia risk more appropriately. We also found that pressure-trained ECTs exhibited greater therapeutic effects than non-trained ECTs at an early stage after transplantation, whereas this difference was no longer evident at later time points. This finding suggests that the therapeutic potential of the cylindrical ECTs used in this study was limited by their ability to cover only a small portion of the injured myocardium. Therefore, the optimization of graft design, including tissue geometry and size, will be important for future regenerative applications.

This approach has the potential to improve the development of functional cardiac tissue models for disease modeling and drug discovery (Ronaldson-Bouchard et al., 2018; Voges et al., 2023). Using iPSC-derived ECTs from patients with fDCM, we found that the maturation-promoting effects of pressure training were limited compared with healthy ECTs, suggesting that these tissues retain disease-specific phenotypes. This finding supports their utility not only for disease modeling but also for drug screening platforms aimed at restoring cardiac function. Because only the protocol optimized for healthy ECTs was tested in this study, alternative pressure intensities or training durations may produce different responses in fDCM-derived tissues. Investigating the effects of various pressure-training regimens on pathological ECTs may provide further insight into how mechanical stimuli influence diseased cardiac tissues and may contribute to the development of novel therapeutic strategies. In addition, the use of gene-corrected isogenic control lines is an important approach for distinguishing mutation-specific effects from donor-to-donor variability (Hnatiuk et al., 2021). We anticipate that this strategy will also be valuable for investigating the genetic mechanisms underlying pressure-induced tissue maturation in future studies.

### Limitations of the study

This study has several limitations. First, the use of a rat model with a high heart rate limited the evaluation of post-transplant arrhythmias and other safety issues. Future studies using large animal models will be necessary to assess the safety and clinical applicability of pressure-trained ECTs. Second, the effects of pressure training under pathological conditions remain incompletely understood. Although fDCM-derived ECTs showed limited maturation responses and may serve as disease models, further studies are needed to determine optimal training conditions for pathological tissues and their potential utility in drug screening. Third, due to technical limitations of the pressure-loading system, pressures below 10 kPa could not be accurately applied. Consequently, whether more physiological loading conditions, such as lower pressures applied over longer durations, can promote maturation remains to be determined. Finally, although pressure training was associated with improved tissue alignment, contractile function, and maturation-related gene expression, causal relationships among these changes have not been established. In addition, histological and molecular analyses were performed using independent sample sets, and some quantitative assessments were based on selected tissue sections rather than whole-tissue analyses. Future studies employing integrated whole-tissue analyses and targeted investigation of EC-specific mechanisms, including vascular network formation and paracrine signaling, will be required to further validate these findings.

## Resource availability

### Lead contact

Further information and request for resources and reagents should be directed and will be fulfilled by the lead contact, Hidetoshi Masumoto (hidetoshi.masumoto@riken.jp).

### Materials availability

This study did not generate new unique reagents.

### Data and code availability

Data supporting the findings of this study are available within the paper and its supplemental information. Primer sequences for qRT-PCR Primer sequences are provided in the key resources table. The single-cell RNA sequencing data generated in this study have been deposited in the Gene Expression Omnibus (GEO): GSE336062 and will be publicly available upon publication. Any additional information required to reanalyze the data reported in this paper is available from the lead contact upon request.

## Acknowledgments

This research was supported by Novartis Global Scholars Program (10.13039/100015311Novartis Institutes for BioMedical Research) (to H.M.), Grant-in-Aid for Scientific Research (17H04291) from the Japan Society for the Promotion of Science (JSPS) (to H.M.), the RIKEN BDR Organoid Project (to H.M.), Research Fellowships for Young Scientists from JSPS (to T.J.), 10.13039/100009619Japan Agency for Medical Research and Development (AMED) (JP22bm1123011 and JP22ama121016) (to S.N.), Grant-in-Aid for Scientific Research (20H03680 and 21K18273) from 10.13039/501100001691JSPS (to W.K.), 10.13039/501100022598Toray Science Foundation (to W.K.), and 10.13039/100016923Hoansha Foundation (to W.K.). The single-cell RNA-seq analysis in this study was supported by 10.13039/100009619AMED BINDS (PI: Dr. Seitaro Nomura, The University of Tokyo, Tokyo, Japan). We thank Dr. Osamu Nishimura and Dr. Takefumi Kondo (RIKEN BDR, Kobe, Japan) for suggestions on single-cell RNA-seq experiments and data analysis. We also thank the Center for Anatomical Studies, Dr. Hirohiko Imai, and Ms. Aiko Yoshioka (Kyoto University, Kyoto, Japan) for the technical support.

## Author contributions

W.M., K. Murata, and H.M. designed research studies. W.M., K. Murata, M.A., L.Y.G.-T., and H.M. conducted iPS cell culture, ECT preparation, pressure training, and histological and functional evaluation experiments and acquired related data. W.M., A.S., Y.S., and W.K. conducted metabolic functional assessment experiments and acquired related data. J.I., K.H., and H.M. conducted animal experiments and acquired related data. W.M., T.J., S.N., and H.M. conducted single-cell RNA-seq experiments and analyzed the data. W.M., J.I., S.T., T.K., and K.Y. conducted collagen alignment evaluations and acquired related data. W.M., L.Y.G.-T., and H.M. conducted statistical analyses. W.M., T.J., T.K., and H.M. wrote the manuscript. K. Minatoya, S.N., W.K., and H.M. supervised this project. All authors reviewed the manuscript.

## Declaration of interests

W.M., K. Murata, and H.M. are inventors of a related patent.

## Declaration of generative AI and AI-assisted technologies in the writing process

During the preparation of this work, the authors used ChatGPT to improve language and readability, with caution. After using this tool/service, the authors reviewed and edited the content as needed and take full responsibility for the content of the publication.

## STAR★Methods

### Key resources table

**Table:** 

REAGENT or RESOURCE	SOURCE	IDENTIFIER
**Antibodies**		

anti-CD144, R-PE, Clone: 55-7H1	BD Biosciences	Catalog # 560410; RRID:AB_1645502
anti-CD140b, R-PE, Clone: 28D4	BD Biosciences	Catalog # 558821; RRID:AB_397132
anti-Troponin T, Cardiac Isoform Ab-1, Clone: 13-11	Thermo Fisher Scientific	Catalog # MS-295-P0; RRID:AB_61807
Human CD31/PECAM-1 Antibody; CD31 (monoclonal mouse IgG1, clone 9G11)	R&D	Catalog # BBA7; RRID:AB_356960
Anti-VE-cadherin-APC (clone 55-7H1)	BD Biosciences	Catalog# 561567; RRID: AB_10712766
Anti-Cardiac Troponin T antibody	Abcam	Catalog# ab45932; RRID: AB_956386
Anti-TOMM20	Abcam	Catalog# ab78547, RRID:AB_2043078
Goat anti-Rabbit IgG (H + L) Cross-Adsorbed Secondary Antibody, Alexa Fluor 488	Thermo Fisher Scientific	Catalog # A-11008; RRID:AB_143165
Goat anti-Mouse IgG (H + L) Cross-Adsorbed Secondary Antibody, Alexa Fluor 546	Thermo Fisher Scientific	Catalog # A- 11003; RRID:AB_2534071

**Chemicals, peptides, and recombinant proteins**		

StemFit AK02N medium	AJINOMOTO	Catalog #AK02N
iMatrix-511 silk	FUJIFILM Wako	Catalog # 387-10131
CultureSure (R) Y-27632 (25mg)	FUJIFILM Wako	Catalog #034-24024
Recombinant human bFGF 100ug	FUJIFILM Wako	Catalog # 060-04543
Human recombinant VEGF 10ug	FUJIFILM Wako	Catalog # 223-01311
Growth factor reduced Matrigel	Corning	Catalog # 356231
Fetal Bovine Serum (FBS)	Corning	Catalog # 35-079-CV
RPMI 1640	Thermo Fisher Scientific	Catalog # 21870092
XF RPMI Medium	Agilent Technologies	Catalog # 103576-100
Seahorse XF Pyruvate Solution	Agilent Technologies	Catalog # 103578-100
Seahorse XF L-Glutamine Solution	Agilent Technologies	Catalog # 103579-100
Isoflurane	Pfizer	N/A
Seahorse XF Glucose Solution	Agilent Technologies	Catalog # 103577-100
Oligomycin	Agilent Technologies	Catalog # 103015-100
FCCP (Carbonyl cyanide-4 (trifluoromethoxy) phenylhydrazone)	Agilent Technologies	Catalog # 103017-100
Rotenone/Antimycin A mixture	Agilent Technologies	Catalog # 103020-100
TrypLE Select	Thermo Fisher Scientific	Catalog # A1285901
B27 supplement minus insulin 10mL	Thermo Fisher Scientific	Catalog # A1895601
Alpha minimum essential medium	Thermo Fisher Scientific	Catalog # 11900024
DAPI (4‘,6-diamidino-2-phenylindole)	Thermo Fisher Scientific	Catalog # D1306
Dulbecco’s modified Eagle’s medium (DMEM)	Thermo Fisher Scientific	Catalog # 11885084
Recombinant Human/Mouse/Rat ActivinA (50μg)	R&D	Catalog # 338-AC-050
BMP4, recombinant (10μg)	R&D	Catalog # 314-BP-010
CHIR99021 10mg	TOCRIS	Catalog # 4423/10
IWP-4 2mg	REPROCELL	Catalog # 04-0036-base
XAV939 10mg	Merck	Catalog # 575545-10MGCN
Accutase	Nacalai Tesque	Catalog # 1267954
Saponin	Nacalai Tesque	Catalog # 30502-42
Penicillin-Streptomycin Mixed Solution	Nacalai Tesque	Catalog # 26253-84
Qiashredder	Qiagen	Catalog # 79654
RNeasy Mini Kit	Qiagen	Catalog # 74104
RNase-Free DNase Set	Qiagen	Catalog # 79254
ReverTra Ace® qPCR RT Master Mix	TOYOBO	Catalog # FSQ-201
PowerUp™ SYBR™ Green Master Mix	Thermo Fisher Scientific	Catalog # A25742
Proteinase K	Qiagen	Catalog # 19131
Pluronic F127	Sigma-Aldrich	Catalog #P2443
High glucose DMEM	Thermo Fisher Scientific	Catalog # 11965092
Collagen Type I	Sigma-Aldrich	
Matrigel	Corning	Catalog # 354234
2-Mercaptoethanol	Sigma-Aldrich	Catalog #M6250
Penicillin-Streptomycin	Thermo Fisher Scientifi	Catalog # 15140122

**Critical commercial assays**		

LIVE/DEAD fixable Aqua dead cell staining kit	Thermo Fisher Scientific	Catalog #L34957
Zenon® APC Mouse IgG1 Labeling Kit	Thermo Fisher Scientific	Catalog #Z25051
*in situ* Apoptosis Detection Kit	Takara Bio Inc	Catalog # MK500
Debris removal solution	Miltenyi Biotec	Catalog # 130-109-398
CalbryteTM 520 a.m.	AAT Bioquest,	Catalog # 20650

**Oligonucleotides**		

human-GAPDH primers	This paper	F: AGAAGGCTGGGGCTCATTTG; R: AGGGGCCATCCACAGTCTTC
human-SERCA2 primers	This paper	F: CTGACGGTGGTCCAAGAGTG; R: CATGGACAGGCAGATGGAGC
human-PLN primers	This paper	F: AAACTCCCCAGCTAAACACC; R: GAACTTCAGAGAAGCATCACGATGATA
human-TNNT2 primers	This paper	F: ACAGAGCGGAAAAGTGGGAAG; R: TCGTTGATCCTGTTTCGGAGA
human-CACNA1C primers	This paper	F: TGATTCCAACGCCACCAATTC; R: GAGGAGTCCATAGGCGATTACT
human-TNNI1 primers	This paper	F: CCGGAAGTCGAGAGAAAACCC; R: TCAATGTCGTATCGCTCCTCA
human-TNNI3 primers	This paper	F: TTTGACCTTCGAGGGCAAGTTT; R: CCCGGTTTTCCTTCTCGGTG
human-MT-ND1 primers	This paper	F: AACCTCAACCTAGGCCTCCT; R: GAGTTTGATGCTCACCCTGA
human-18S5 primers	This paper	F: GCTGAGAAGACGGTCGAACT; R: CGCAGGTTCACCTACGGAAA

**Experimental models: Cell lines**		

Human iPSCs lines 201B6	the Center for iPS Cell Research and Application (CiRA), Kyoto University, Kyoto, Japan	N/A
Human iPSCs lines FF101s04	the Center for iPS Cell Research and Application (CiRA), Kyoto University, Kyoto, Japan	N/A
HPS1799	National BioResource Project	N/A

**Software and algorithms**		

CytExpert software	Beckman Coulter	N/A
BZ-X800E sectioning functions	Keyence	N/A
MUSCLEMOTION	Open source	N/A
ImageJ	Open source	N/A
Prism 9	GraphPad	N/A
Chromium Single Cell Platform	10*x* Genomics	N/A
Scanpy	Scanpy	N/A
Stata Statistical Software	StataCorp	N/A

**Deposited data**		

Single-cell RNA sequencing data	This paper	GEO: GSE336062

**Other**		

Compact Digital Rocker	DLAB	Catalog # 3-7044-09
CytoFLEX S	Beckman Coulter,	N/A
Electrical pulse generator	Strex, Osaka, Japan	N/A
Electron microscope	H-7650; Hitachi	N/A
Seahorse XFp Analyzer	Agilent	Catalog #S7802A
NanoDrop One Spectrophotometer	Thermo Fisher Scientific	N/A
Applied Biosystems StepOne Plus Real-Time PCR System	Thermo Fisher Scientific	N/A
Zeiss LSM880	ZEISS	N/A
MechanoCulture TR Stimulator	CellScale	N/A

### Experimental model and study participant details

All animal experiment protocols were approved by the Animal Experimentation Committee of Kyoto University (#MedKyo 23155). All animal experiments were conducted in accordance with Japanese law and the “*Guide for the Care and Use of Laboratory Animals*” of the US National Research Council. Male athymic nude rats (F344/N Jcl-rnu/rnu; CLEA Japan, Tokyo, Japan) aged 10–13 weeks were used for transplantation experiments. Animals were housed under specific pathogen-free conditions at the Institute of Laboratory Animals, Graduate School of Medicine, Kyoto University, in a temperature-controlled environment (24 ± 2°C) with a 12-h light/12-h dark cycle and *ad libitum* access to food and water.

#### Sex as a biological variable

We used only male rats as the animal model in this study to avoid the influence of endocrine factors on cardiac function outcomes. It is unknown whether the findings are relevant for female rats.

### Method details

#### Human iPSC culture and differentiation

Three human iPSC lines were used in this study: 201B6 [4-factor (Oct3/4, Sox2, Klf4, and c-Myc)] (Takahashi et al., 2007), FFI01s04 line (Minagawa et al., 2018), which is a human leukocyte antigen homozygous iPSC line established at the Center for iPS Cell Research and Application (CiRA), Kyoto University, Kyoto, Japan, and distributed subject to the informed consent of a healthy donor and the permission of the Institutional Review Board (G1025), and HPS1799 [derived from a familial dilated cardiomyopathy patient donor; provided by the RIKEN BRC through the National BioResource Project of the MEXT, Japan; approved by RIKEN Institutional Review Board (Kobe1 2020–06)] (https://cell.brc.riken.jp/en/diseaselist_5). The culture and differentiation of human iPSCs into cardiovascular cells were performed following previously established protocols, with some modifications (Murata et al., 2024). Briefly, iPSCs were expanded and maintained in StemFit AK02N medium (AJINOMOTO, Tokyo, Japan) under feeder-free conditions. The cells were passaged at 80% confluency using TrypLE Select (Thermo Fisher Scientific, Waltham, MA, USA), then resuspended in a solution containing 0.5 mM ethylenediaminetetraacetic acid in phosphate-buffered saline (PBS) (1:1), along with AK02N medium supplemented with iMatrix-511 silk (0.125 μg/cm^2^, FUJIFILM Wako Pure Chemical Corp., Osaka, Japan) (Miyazaki et al., 2017) and the ROCK inhibitor Y-27632 (10 μM, FUJIFILM Wako).

For cardiovascular cell differentiation, 3.6 to 4 million iPSCs/well were seeded onto Matrigel-coated 6-well plates (Matrigel, Corning, New York, NY, USA; Falcon, Corning) in AK02N medium supplemented with Y-27632 (10 μM). Once the cells reached confluence, they were covered with Matrigel (1:60 dilution in AK02N) one day before beginning the differentiation process. The differentiation process began with replacing the AK02N medium with RPMI + B27 medium (RPMI 1640, Thermo Fisher; 2 mM L-glutamine, Thermo Fisher; 1× B27 supplement without insulin, Thermo Fisher) containing 100 ng/mL Activin A (R&D, Minneapolis, MN, USA) and 5 μM CHIR99021 (Tocris Bioscience, Bristol, UK) on differentiation day 0 (d0) for 24 h. On d1, the medium was supplemented with 10 ng/mL bone morphogenetic protein 4 (BMP4; R&D) and 10 ng/mL basic fibroblast growth factor (bFGF), with this medium being maintained for 4 days without change. On d5, the medium was adjusted to induce either the formation of both cardiomyocytes (CM) and endothelial cells (EC) (CM + EC protocol) or cardiomyocytes alone (CM protocol). For the CM + EC protocol, the medium was switched to RPMI1640 supplemented with 50 ng/mL VEGF (FUJIFILM Wako), 2.5 μM IWP4 (Stemgent, Cambridge, MA, USA), and 5 μM XAV939 (Merck, Kenilworth, NJ, USA), and refreshed every other day with RPMI1640 minus insulin and 50 ng/mL VEGF. In contrast, for the CM protocol, the medium on d5 was replaced with RPMI + B27 (RPMI 1640; 2 mM L-glutamine; 1× B27 insulin supplement) containing 2.5 μM IWP4 and 5 μM XAV939 and refreshed every other day with RPMI1640 plus insulin. Beating cells appeared between d11 and d13. To regulate the percentage of mural cells (MCs) necessary for forming engineered cardiac tissue (ECT), the mural cell differentiation (MC protocol) was initiated on d0 using the same steps as other 2 protocols, except for excluding CHIR99021. From d3 onwards, the medium was changed to RPMI + FBS medium [RPMI 1640 with 2 mM L-glutamine and 10% fetal bovine serum (FBS)], and this medium was refreshed every other day.

#### Flow cytometry

On d13, cells from the CM + EC protocol, CM protocol, and MC protocol were dissociated using Accutase (Nacalai Tesque, Kyoto, Japan). A total of 500,000 cells were collected from each protocol for subsequent flow cytometry analysis. Dead cells were excluded by staining with the LIVE/DEAD Fixable Aqua Dead Cell Staining Kit (Thermo Fisher). The cells were then labeled with specific surface markers to identify different cell types. For intracellular proteins, cells were fixed with 4% paraformaldehyde (PFA) in PBS before staining. Mural cells were identified using anti-PDGFRβ conjugated with phycoerythrin (PE) (clone 28d4, 1:100, BD, Franklin Lakes, NJ, USA), endothelial cells with anti-VE-cadherin conjugated with allophycocyanin (APC) (clone 55-7h1, 1:100, BD), and cardiomyocytes with anti-cardiac troponin T (cTnT) (clone 13–11, Thermo Fisher), which was labeled with APC using Zenon technology (Thermo Fisher) at a 1:50 dilution. The stained cells were analyzed using a CytoFLEX S flow cytometer (Beckman Coulter, Brea, CA, USA), with data processed in CytExpert software (Beckman Coulter).

#### Tissue mold fabrication

Tissue molds were fabricated using polydimethylsiloxane (PDMS) (Strex, Osaka, Japan) (Figure S1). The PDMS molds were autoclaved before use. After sterilization, the molds were treated with 1% Pluronic F127 (Sigma-Aldrich, St. Louis, MO, USA) for 1h to prevent cell attachment to the mold surface. Once the coating period was complete, the Pluronic F127 solution was removed, and the molds were thoroughly rinsed with PBS. The molds were then prepared and ready for tissue molding.

#### ECT generation

To generate ECTs with or without ECs, differentiated cells from the CM + EC or CM protocols were combined with cells from the MC protocol, ensuring a final MC concentration of 10%–15%. We adapted our previous methods for this process. Based on flow cytometry results, CM, EC, and MC were mixed to achieve the desired MC concentration. A total of six million cells were used for each ECT type: ECT with EC (CM + EC + MC) and ECT without EC (CM + MC). These cells were suspended in a culture medium composed of high glucose Dulbecco’s Modified Essential Medium (DMEM) (Life Technologies) supplemented with 20% fetal bovine serum (Life Technologies). The matrix solution was prepared with acid-soluble collagen type I (Sigma, 2 mg/mL, pH 3), an alkali buffer (0.2M NaHCO_3_, 0.2M HEPES, 0.1M NaOH), and Matrigel (BD, 16.7% of the total volume). The cell suspension and matrix solution were mixed to reach a final collagen type I concentration of 0.67 mg/mL in a total volume of 200 μL. This mixture was then poured into Pluronic F127-coated PDMS molds, which were placed in a twelve-well culture plate and allowed to polymerize in a CO_2_ incubator at 37°C with 5% CO_2_ for 60 min. Following polymerization, the molds were immersed in ECT medium [alpha minimum essential medium (αMEM; Life Technologies) supplemented with 10% FBS, 0.05 mM 2-mercaptoethanol (Sigma), and 100 U/mL Penicillin-Streptomycin (Life Technologies)]. The ECTs were cultured for 14 or 21 days, with the medium changed every other day.

#### Pressure stimulation

Hydrostatic pressure stimulation was performed in a MechanoCulture TR (MCTR) stimulator (CellScale, Waterloo, ON, Canada) (Swiatlowska et al., 2022), placed inside a tissue culture incubator. Tissue mold with ECT were moved to MCTR chamber with filling up medium, set several pressure stimulation protocol (Continues pressure, 10 kPa, 50 kPa and 100 kPa) (compress: 0.2s, hold: 0.1s, recovery: 0.2s, rest: 0.5s) following the instruction of the manufacturer. After hydrostatic pressure stimulation, ECTs were returned to the culture plate and kept in culture.

We applied hydrostatic pressure stimulation to ECTs by modulating the pressure within a sealed culture chamber. This method alters the surrounding pressure environment without directly stretching or compressing the tissue structure. Therefore, the mechanical stimulus used in this study represents hydrostatic pressure loading rather than tensile stretch or mechanical deformation of the tissue.

#### Histological analysis and fluorescence assay

ECTs were fixed in 4% PFA for 30 min and embedded in paraffin. Sections (6-μm thickness) were prepared and stained with hematoxylin–eosin, Sirius red (SR), cTnT, and TdT-mediated dUTP Nick-End Labeling (TUNEL). For fluorescence microscopy, ECTs were stained for cTnT (Abcam, ab45932) and CD31 (monoclonal mouse IgG1, clone 9G11) (R&D) (1:400) with DAPI (4′,6-diamidino-2-phenylindole) (Thermo Fisher), the single cells were stained for cTnT (Mouse Monoclonal Antibody, MS-295-P1) (1:400), TOMM20 (Abcam, ab78547) (1:400) with DAPI (4′,6-diamidino-2-phenylindole) (Thermo Fisher), Anti-mouse Alexa 546 (Thermo Fisher) and anti-rabbit Alexa 488 (Thermo Fisher) were used as secondary antibodies. The tissues or single cells were photographed with an all-in-one fluorescence microscopic system, Zeiss LSM880 (MFI of TOMM20 by ImageJ) (Schneider et al., 2012).

To evaluate the alignment of collagen, polarized light microscopy was performed on SR-stained samples by using the microscope with a 5× objective lens (Eclipse LV100N POL, Nikon, Tokyo, Japan). The alignment was assessed based on the difference in brightness observed when the angle between the transmission axis of the polarizer and the optical axis of the sample was set to 0° and 90°.

Quantitative analysis of ECT, including cell number, cTnT positive area, cell size, cell perimeter, TOMM20 mean fluorescence intensity (MFI), thickness changing of ECT with EC or without EC over the time were analyzed by ImageJ software.

#### Electrical stimulation test

Before the initial hydrostatic pressure stimulation and after the final stimulation, we conducted electrical stimulation of the ECTs using a custom-built electrical stimulation system connected to an electrical pulse generator (Strex, Osaka, Japan). The tissues were exposed to electrical pulses at 30 V (peak-to-peak) with a pulse width of 4 ms. The stimulation frequency started at an interval of 1000 ms (1 Hz) and gradually increased to 500 ms (2 Hz). The response of the ECTs to the electrical stimulation was monitored and verified through real-time microscopic observations. Motion amplitude was normalized to the baseline value at a stimulation interval of 1000 ms (1 Hz) for each individual ECT. Specifically, motion amplitudes measured at shorter stimulation intervals (e.g., 900 ms, 800 ms, 700 ms) were divided by the corresponding amplitude at 1000 ms within the same sample. This normalization ensured that all datasets started from a value of 1.0 at 1 Hz, allowing for comparison of relative changes in contractility in response to increasing stimulation frequency. A progressive increase in normalized motion amplitude with increasing stimulation frequency was interpreted as a positive force–frequency relationship (FFR).

#### MUSCLEMOTION analysis

MUSCLEMOTION is an open-source software that utilizes a video-based system to assess contractile functions (Sala et al., 2018). We adhered to the provider’s guidelines for using the software. Briefly, MUSCLEMOTION was installed as a plug-in within ImageJ software, and the motion amplitude was used as the key parameter for analysis.

#### Calcium transient assay

CalbryteTM 520 a.m. (AAT Bioquest, Pleasanton, CA, USA; final concentration of 5 μM) loading solution, containing 0.04% PF-127, was added to the tissue molds. The ECTs were incubated with the dye-loading buffer for 60 min in a 5% CO_2_ incubator at 37°C. After incubation, the dye-loading solution was replaced with FluoroBriteTM DMEM for analysis. Videos of the signal intensity were captured using fluorescence microscopy (CKX53, Evident, Tokyo, Japan) equipped with a FITC filter, a camera system (DP27), and software (cellSens, Evident). Data were analyzed using ImageJ software, with manually selected regions of interest (ROIs) and the “Plot z axis Profile” function.

#### Transmission electron microscopy

Sample were fixed in 2% glutaraldehyde+4% PFA/0.1M phosphate buffer (pH 7.2) at 4°C overnight and sent to the Center for Anatomical Studies, Kyoto University (Kyoto, Japan) for subsequent sample preparation and imaging as previously reported (Setozaki et al., 2017). In brief, the tissue samples were cut into ultra-thin sections on an ultramicrotome and observed under an electron microscope (H-7650; Hitachi, Tokyo, Japan). Analyses were made in a blinded manner.

#### ECT dissociation and debris removal procedure

After ECT were washed twice using PBS, incubate the ECT on a rotator for 1.5–2 h at 37°C with Collagenase Type 2 (2 mg/mL Worthington) in dissociation buffer [120 mM NaCl/5.4 mM KCl/5 mM MgSO_4_/5 mM Na-Pyruvate/20 mM Glucose/20 mM Taurine/10 mM HEPES (pH 6.9)/30 μM CaCl_2_], then dissociated tissue were performed debris removal experiment using debris removal solution (Miltenyi Biotec, Bergisch Gladbach, Germany), was used according to the manufacturer’s instructions as follows, (a) Centrifuge the cell suspension at 300×g for 10 min at 4°C. (b) Aspirate supernatant completely and resuspend cell suspension with the 500 μL cold PBS, add same volume of cold debris removal solution, then overlay very gently with cold PBS (c) Centrifuge at 4°C and 3000×g for 10 min with full acceleration and full brake. Three phases are formed, aspirate the two top phases completely and discard them and fill up with cold PBS, gently invert the tube three times. (d) Centrifuge at 4°C and 1000×g for 10 min with full acceleration and full brake, then aspirate supernatant completely.

#### Cellular metabolism assay

After dissociation of ECT and debris removement described above, the purified single iPSC-CMs were seeded onto Matrigel-coated Seahorse XFp assay plates (Agilent, Santa Clara, CA, USA) at a density of 100,000 cells/well and allowed to grow for 3 days with ECT medium. One hour prior to the assay, the plates were changed to Seahorse assay media (XF RPMI supplemented with 10 mM glucose, 1 mM pyruvate, and 2 mM Glutamine), then incubated at 37°C in a CO_2_-free incubator with Seahorse assay media. The XF Cell Mito Stress Test Kit was used according to the manufacturer’s instructions, with the following final concentrations for the injected compounds: 3 μM oligomycin, 2 μM 2-[2-[4-(trifluoromethoxy)phenyl]hydrazinylidene]-propanedinitrile (FCCP), and 2 μM rotenone/antimycin A.

#### Quantitative real-time PCR (RT-PCR)

Total RNA was extracted using the Qiashredder (Qiagen, Hilden, Germany) and subsequently purified with the RNeasy Mini Kit (Qiagen), following the manufacturer’s guidelines for both procedures. The purification process included a DNase treatment using the RNase-Free DNase Set (Qiagen). RNA yield and purity were assessed with a NanoDrop One spectrophotometer (Thermo Fisher). First-strand cDNA was synthesized from 200 ng of total RNA for each sample using the ReverTra Ace qPCR RT Master Mix (FSQ-201, TOYOBO, Osaka, Japan) as per the manufacturer’s instructions. RNA was then analyzed by quantitative real-time PCR (RT-PCR) using the Applied Biosystems StepOne Plus system (Thermo Fisher) with the PowerUp SYBR Green Master Mix (Thermo Fisher), following the manufacturer’s protocol. Data analysis was performed using the fold change method, normalized to the expression of the glyceraldehyde-3-phosphate dehydrogenase (*GAPDH*) gene.

The MtDNA was extracted from ECT using the DNA digestion buffer (1:100 of Proteinase K to Qiagen Cell Lysis solution). MtDNA was analyzed by quantitative RT-PCR in Applied Biosystems StepOne Plus (Thermo Fisher) using the PowerUpTM SYBR Green Master Mix (Thermo Fisher) according to the manufacturer’s instructions. Primers for MtDNA was mitochondrial gene NADH dehydrogenase 1 (*ND1*) and normalized to *RNA18S5* gene expression. Primer sequences are provided in the Key Resources Table.

#### Single-cell RNA-seq

After dissociation of ECT and debris removement described above, the purified single cells were subjected to single-cell RNA-seq experiments using the Chromium Single-Cell platform (10*x* Genomics, Pleasanton, CA, USA). Single-cell RNA-seq libraries were prepared using the 10*x* Genomics Chromium Next GEM Single Cell 3′ Reagent Kits v3.1, following the standard protocol for Dual Index library preparation. After cDNA synthesis, libraries were assessed for quality using TapeStation and quantified by Qubit and qPCR. The final library concentration was adjusted to 300 pM for sequencing. Libraries were sequenced on the Illumina NovaSeq 6000 using the standard 10*x* Genomics 28-10-10-90 cycle workflow, yielding high-quality data with a depth of 50,000 reads per cell.

#### Data analysis of single-cell RNA-seq

Raw FASTQ files were processed using the Cell Ranger software (version 7.1.0, 10*x* Genomics), against the hg 38 reference genome ‘GRCh38-2020-A’ with the default setting. Python (v.3.10), Pandas (v.2.2.2), NumPy (v.1.26.4), and Scanpy (v.1.10.2) were used for quality control and downstream processing. Genes detected in less than 3 cells were filtered. In addition, cells were filtered for genes (3000 < n_counts <12,000) and mitochondrial genes (percent_mito <20%). We performed downstream processing, including normalization (normalize_per_cell: counts_per_cell_after = 10,000), log transformation (log1p), variable gene detection (highly_variable_genes, *n* = 5000), scaling (scale: max_value = 10) and PCA (using highly variable genes) (Luecken and Theis, 2019). Neighbors were estimated using (n_neighbors = 10 and n_pcs = 40), and we performed Leiden clustering (resolution = 0.2) and UMAP visualization for identifying subpopulations and visualization. We calculated differentially expressed genes using the Wilcoxon rank-sum test, and genes were ranked by score. The identified clusters were annotated based on the expression of major marker genes and differentially expressed genes across the clusters.

#### Subpopulation analysis of single-cell RNA-seq

Subpopulation analysis was performed for clusters annotated as CM. We recalculated PCA, neighbors, and UMAP and visualized the cells. Differentially expressed genes across the conditions were determined using the Wilcoxon rank-sum test. GO enrichment analysis was performed using gseapy using the top 100 ranked genes for each condition with GO_Biological_Process_2021 (Fang et al., 2023). Top 10 enrichment terms were selected for visualization using the -log10(Adjusted *p*-value). Gene score was calculated for each cell with the score_genes function implemented in Scanpy. Lists of genes were collected using the gene set of “GOBP_CARDIAC_MUSCLE_CONTRACTION.v2023.2” for cardiac contraction score, “GOBP_REGULATION_OF_CARDIAC_MUSCLE_CONTRACTION_BY_CALCIUM_ION_SIGNALING.v2023.2” for calcium signaling score, “GOBP_GENERATION_OF_PRECURSOR_METABOLITES_AND_ENERGY” for metabolites energy score, and “GOBP_CARDIAC_CHAMBER_DEVELOPMENT.v2023.2” for cardiac development score with the subsequent visualization and the comparison across the conditions (Subramanian et al., 2005).

#### Animal model preparation and ECT transplantation

All animal experiment protocols were approved by the Animal Experimentation Committee of Kyoto University (#MedKyo 23155). All animal experiments were conducted in accordance with Japanese law and the “Guide for the Care and Use of Laboratory Animals” of the US National Research Council. Male athymic nude rats (F344/N Jcl-rnu/rnu, CLEA Japan, Inc., Tokyo, Japan), aged 10–13 weeks, were utilized for transplantation experiments. For the experiment shown in Figure 5, FfI01s04 iPS cell line was used. The rats were randomly assigned to two groups: one group received ECTs that had undergone pressure training (treatment group), while the other group underwent a sham operation (sham group). For the experiment shown in Figure S14, 201B6 iPS cell line was used. the rats were randomly assigned to three groups: one group received ECTs that had undergone pressure training (treatment group), one group received ECTs without pressure training (Control group), while the other group underwent a sham operation (sham group). Under general anesthesia using continuous inhalation of 1.5–2.5% isoflurane (Pfizer, New York, NY, USA), myocardial infarction (MI) models were generated in the rats by the permanent ligation of left anterior descending artery through left thoracotomy as previously described (Osada et al., 2021). One week later, The ECTs were secured to the surface of the heart using the method previously reported (Masumoto et al., 2016), following a second left thoracotomy. Three ECTs were transplanted into the heart of each rat. Echocardiogram (Echo) assessments were conducted on the day of MI induction (day −7), on the day of ECT transplantation (day 0), and again two weeks post-transplantation (day 14). Four weeks after transplantation (day 28), magnetic resonance imaging (MRI) and echocardiography were performed, followed by the harvesting of the heart after euthanasia.

#### Echocardiogram

Echocardiogram was conducted using the Vivid 7 system (GE Healthcare, Waukesha, WI, USA) equipped with an 11-MHz imaging transducer (GE 10S ultrasound probe, GE Healthcare) in a double-blinded manner. End-diastolic dimension of LV (LVDd), End-systolic dimension of LV (LVDs), and End-diastolic volume of LV (LVEDV) were measured using M-mode examination. Fractional shortening (FS) was calculated as follows: FS (%) = (LVDd - LVDs)/LVDd × 100.

#### Cardiac MRI

At the end of the 4-week observation period, under general anesthesia with 1.5–2.5% isoflurane (Pfizer), MRI (7-T BioSpec 70/20 USR; Bruker Biospin, Ettlingen, Germany) was used for cardiac functional evaluation. LVEDV (left ventricular end-diastolic volume) and LVESV (left ventricular end-systolic volume) were obtained using ImageJ software. LVEF (left ventricular ejection fraction) was calculated using the following formula: LVEF (%) = 100 × (LVEDV - LVESV)/(LVEDV).

### Quantification and statistical analysis

Continuous data are presented as mean ± standard deviation (SD). Normality was assessed by visual inspection of boxplot distributions and then confirmed with Shapiro–Wilk test, due to sample size. Based on these assessments, either parametric or nonparametric tests were applied. Statistical comparisons of normally distributed data were conducted using unpaired t-tests for independent group comparisons and a repeated measures ANOVA was performed to account for the within-sample variability and assess the differences in contractile force across the different stimulation frequencies. An alpha level of 0.05 was set as the threshold for statistical significance for all tests, considering *p*-value <0.05 as statistically significant. All analyses were performed using GraphPad Prism version 9.0 software (GraphPad Software, Boston, MA, USA) and Stata Statistical Software: Release 18 (StataCorp, College Station, TX, USA).

## Supplemental information

Document S1. Figures S1–S16

Document S2. Article plus supplemental information
