# Supplementary material for: High and intermittent hydrostatic pressure promotes maturation of engineered cardiac tissues derived from human pluripotent stem cells
Source: Stem Cell Reports. 2026 Jul 23;21(8):103019. doi: 10.1016/j.stemcr.2026.103019 (PMC13476875; doi:10.1016/j.stemcr.2026.103019)
Supplement: Supplementary file 1 [file mmc1.pdf]

Stem Cell Reports, Volume *21*

Supplemental Information

High and intermittent hydrostatic pressure promotes maturation of engineered cardiac tissues derived from human pluripotent stem cells

Wusiman Maihemuti, Kozue Murata, Takahiro Jimba, Jun Iida, Keisuke Hakamada, Akane Sakaguchi, Mosha Abulaiti, Yuichi Saito, Laura Yuriko González-Teshima, Satoshi Takatori, Takahiro Kenmotsu, Kenichi Yoshikawa, Kenji Minatoya, Seitaro Nomura, Wataru Kimura, and Hidetoshi Masumoto

Supplemental Information

High and intermittent hydrostatic pressure promotes maturation of engineered cardiac tissues derived from human pluripotent stem cells

Wusiman Maihemuti, Kozue Murata, Takahiro Jimba, Jun Iida, Keisuke Hakamada, Akane Sakaguchi, Mosha Abulaiti, Yuichi Saito, Laura Yuriko González-Teshima, Satoshi Takatori, Takahiro Kenmotsu, Kenichi Yoshikawa, Kenji Minatoya, Seitaro Nomura, Wataru Kimura, and Hidetoshi Masumoto

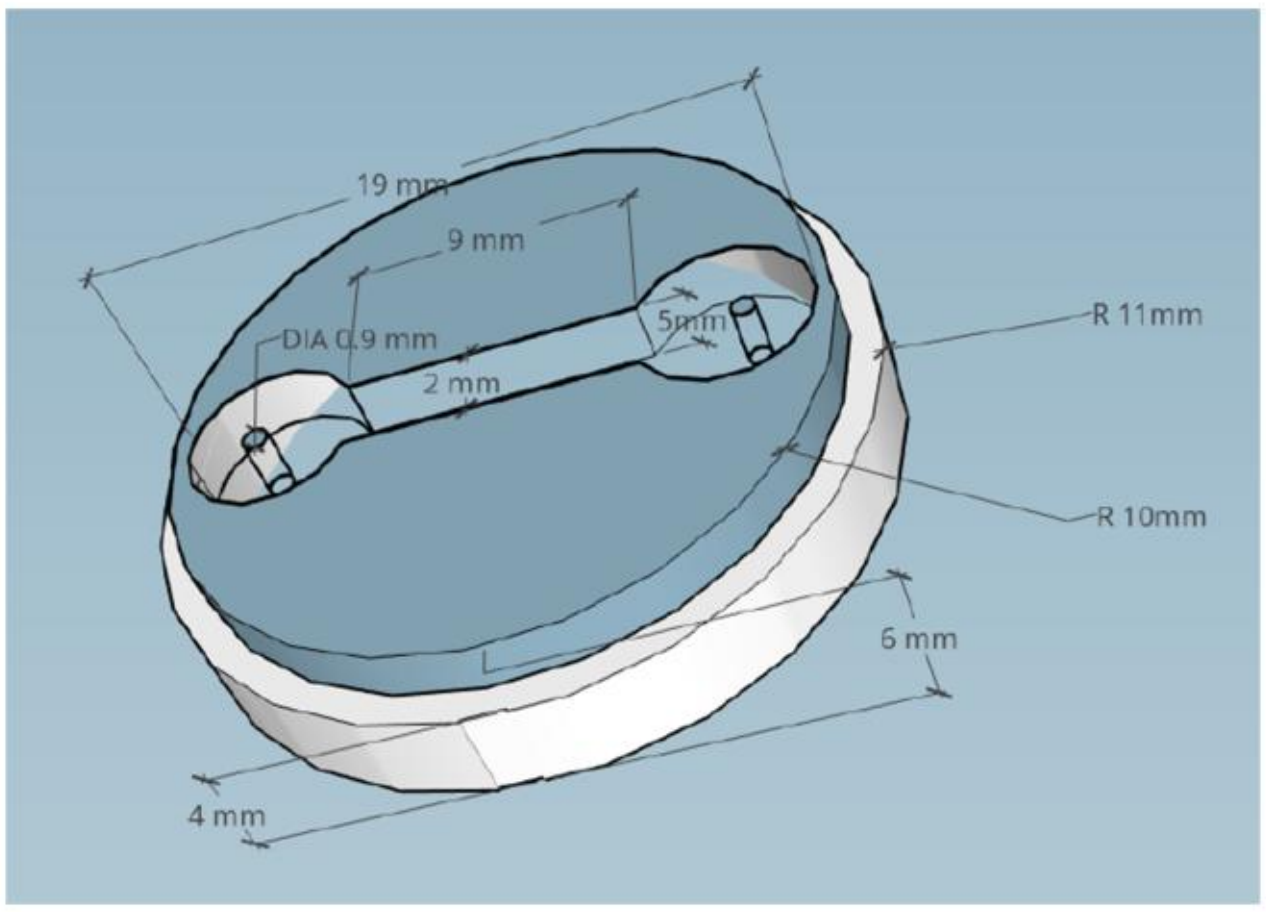

Figure S1: The design of tissue mold fabricated by polydimethylsiloxane (PDMS)

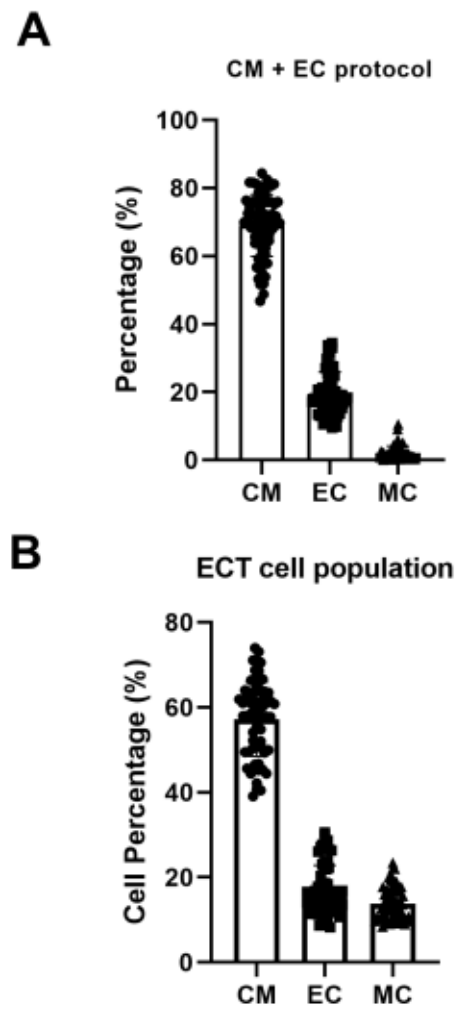

Figure S2: Cellular components. (A) Cellular components of each differentiation protocol. CM: cardiomyocytes; EC: vascular endothelial cells; MC: vascular mural cells. **(B)** Cellular components of all generated engineered cardiac tissues (ECTs). For (A) and (B), n = 57 independent measurements (ECT-based; each derived from independent differentiation experiments).

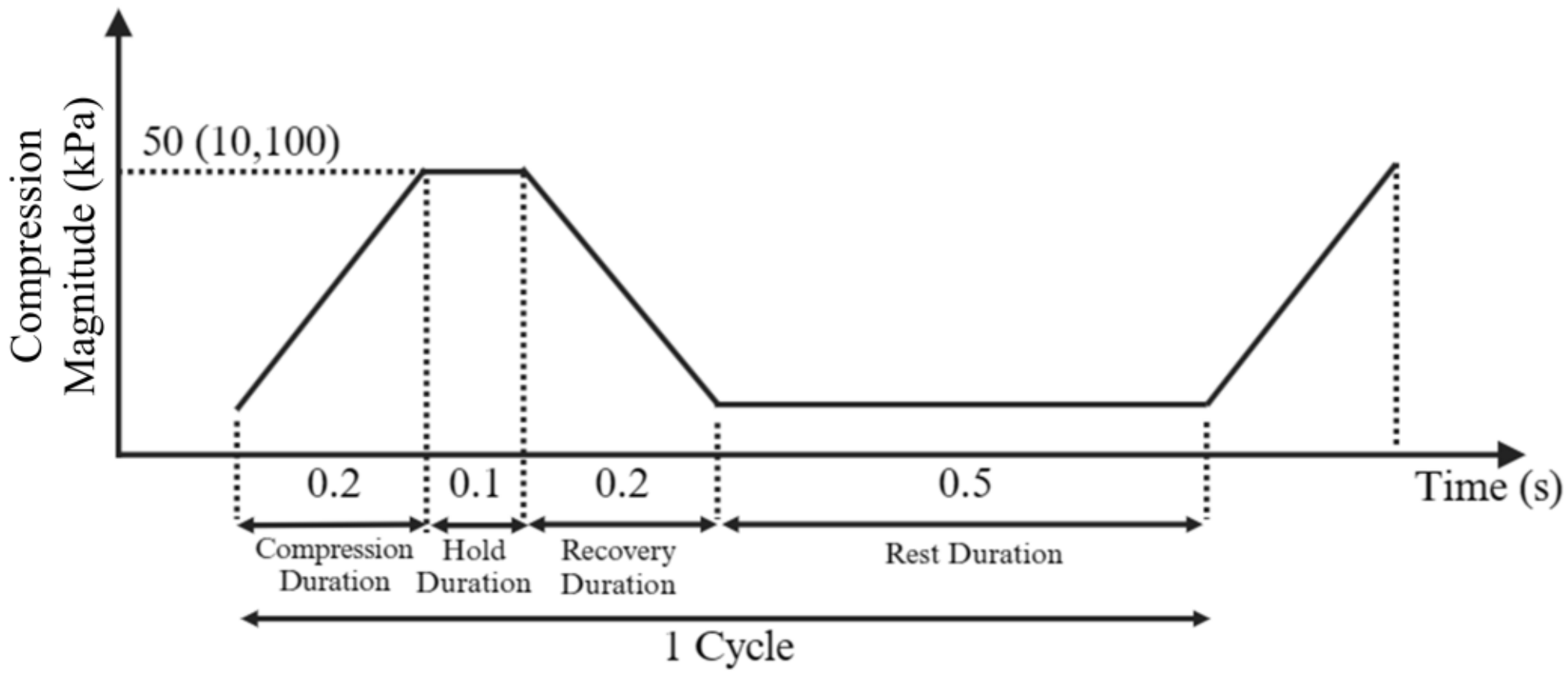

Figure S3: Hydrostatic pressure loading pattern. The figure illustrates the temporal pattern of pressure application within the culture chamber. This figure is intended to demonstrate the loading schedule and does not represent tissue compression or tensile deformation.

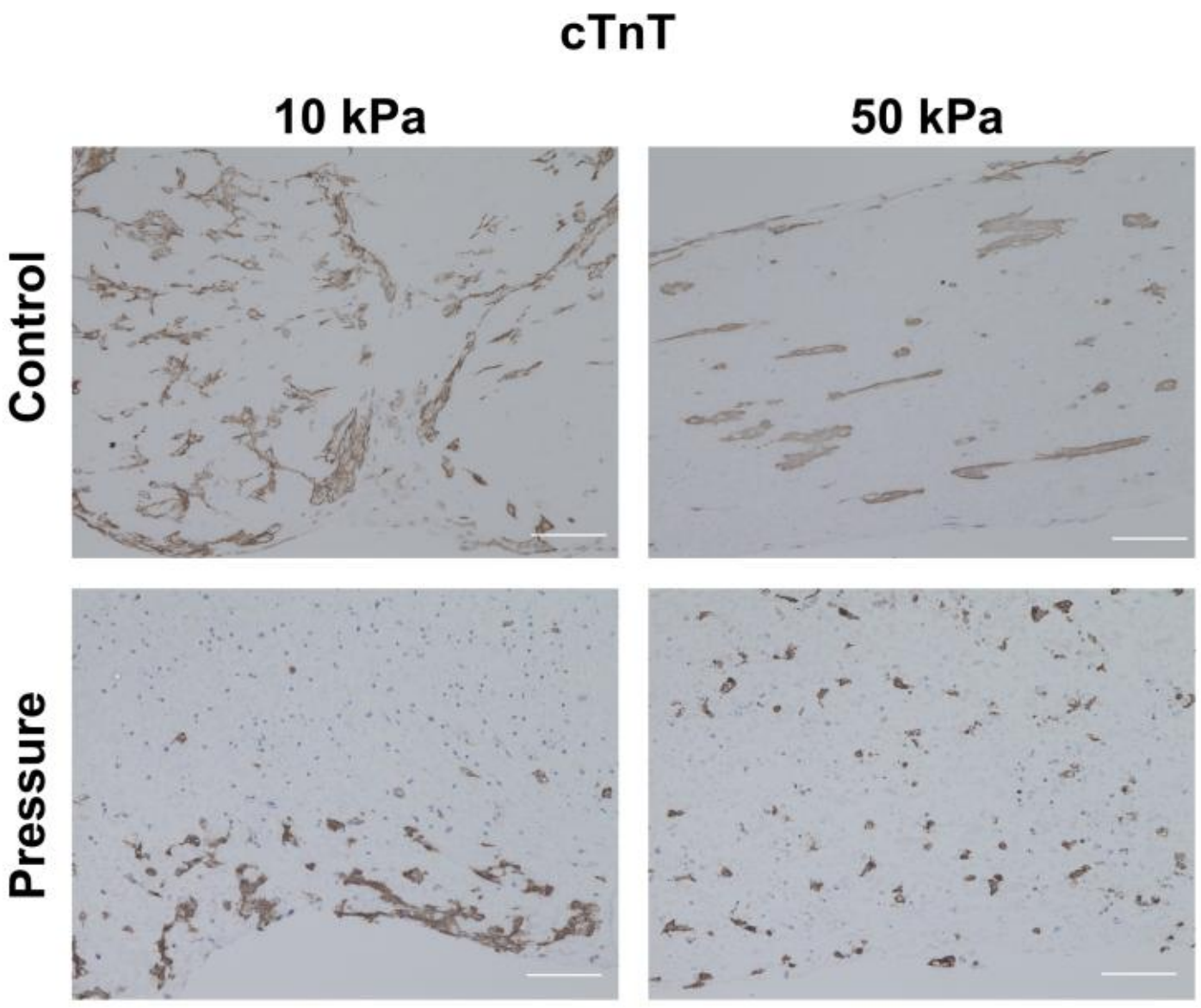

Figure S4: Histological staining of cardiac isoform of troponin-T (cTnT) under continuous hydrostatic pressure in 10kPa and 50 kPa. N = 3 independent ECTs derived from independent differentiation experiments. Scale bars = 100 µm.

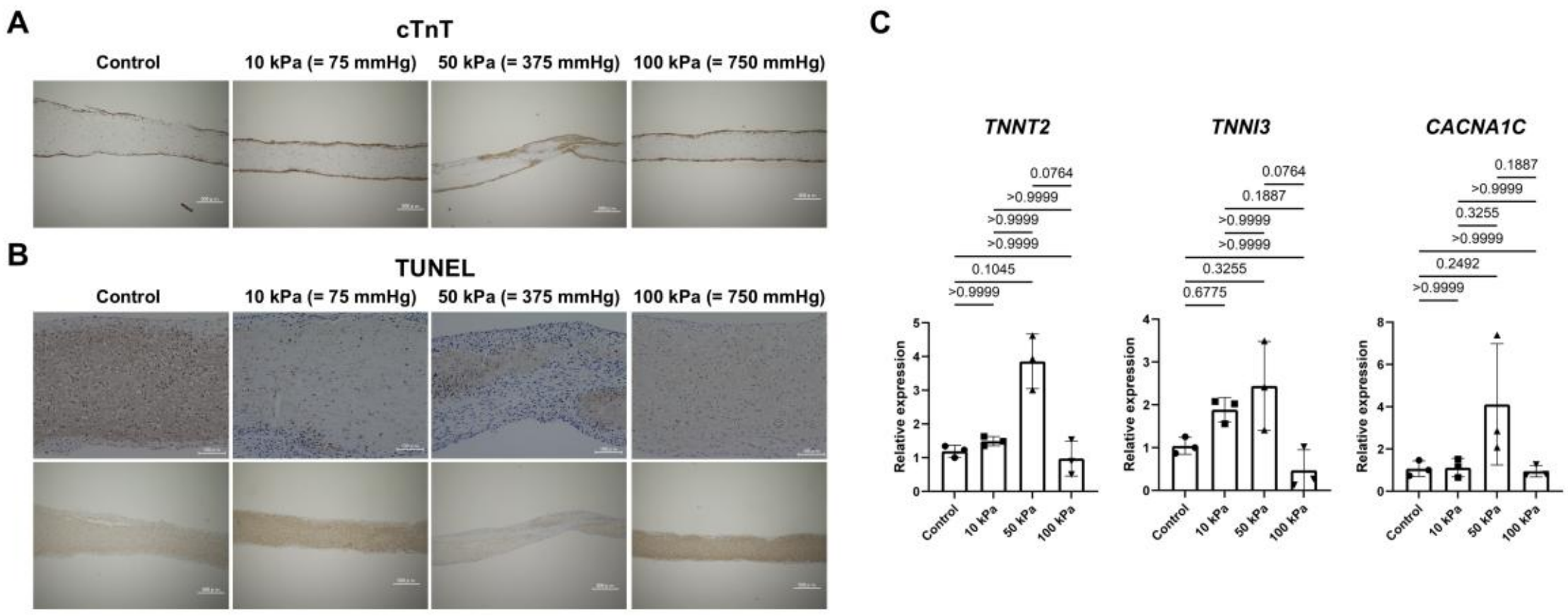

Figure S5: Intermittent training under different hydrostatic pressure. (A) Representative immunostaining of ECTs for cTnT (lower magnification image of Figure 1C). Scale bars = 500 µm. **(B)** Representative terminal deoxynucleotidyl transferase dUTP nick end labeling (TUNEL) staining of ECTs. Scale bars = 100 µm (high magnification; upper), 500 µm (low magnification; lower). **(C)** RT-qPCR. TNNT2: cardiac troponin T2, TNNI3: troponin I3; CACNA1C: calcium voltage-gated channel subunit alpha1 C. For (A) to (C), n = 3 independent ECTs derived from independent differentiation experiments.

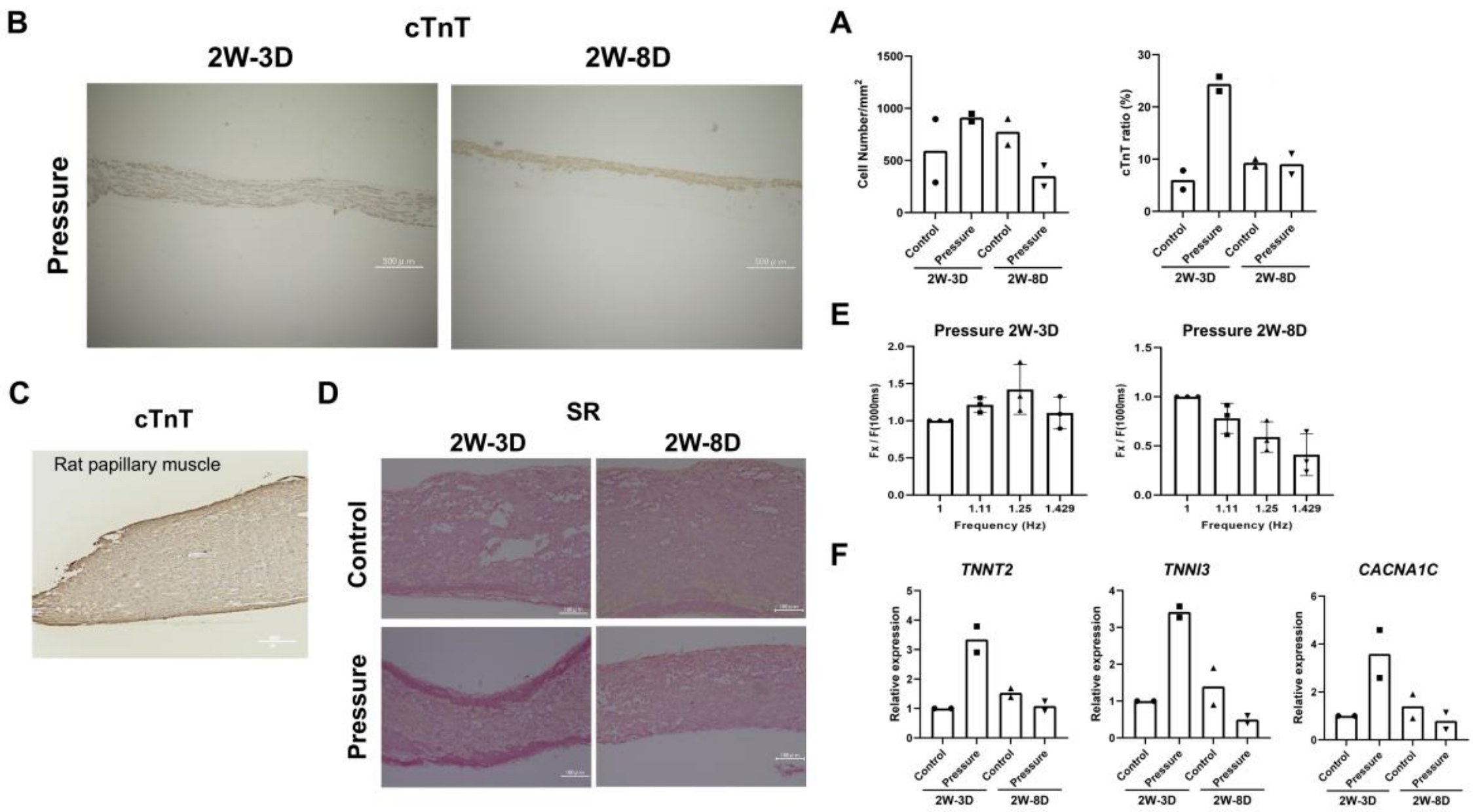

Figure S6: Intermittent training under different pressure stimulation duration. (A) Quantitative analysis for cell number (/mm2) evaluated by hematoxylin and eosin staining (left) and cTnT positive ration among whole tissue area evaluated by cTnT immunostaining (right). N = 2 independent ECTs derived from independent differentiation experiments. **(B)** Representative immunostaining for cTnT. 2W-3D: pre-maturation 2 weeks and stimulation 3 days; 2W-8D: pre-maturation 2 weeks and stimulation 8 days. N = 7 independent ECTs derived from independent differentiation experiments. Scale bars = 500 μm. **(C)** cTnT immunostaining of rat papillary muscle. Scale bar = 500 μm. **(D)** Representative Sirius red staining (SR) of ECTs. 2W-3D: pre-maturation 2 weeks and stimulation 3 days; 2W-8D: pre-maturation 2 weeks and stimulation 8 days. N = 7 independent ECTs derived from independent differentiation experiments. Scale bars = 100 μm. **(E)** Quantified changes in the motion amplitude in accordance with frequency of external electrical stimulation. Fx represents F(1000ms), F(900ms), F(800ms) and F(700ms), correspondingly. N = 3 independent ECTs derived from independent differentiation experiments. **(F)** RT-qPCR. *TNNT2*: cardiac troponin T2; TNNI3: troponin I3; CACNA1C: calcium voltage-gated channel subunit alpha1 C. N = 2 independent ECTs derived from independent differentiation experiments. Data are presented as mean ± standard deviation (SD).

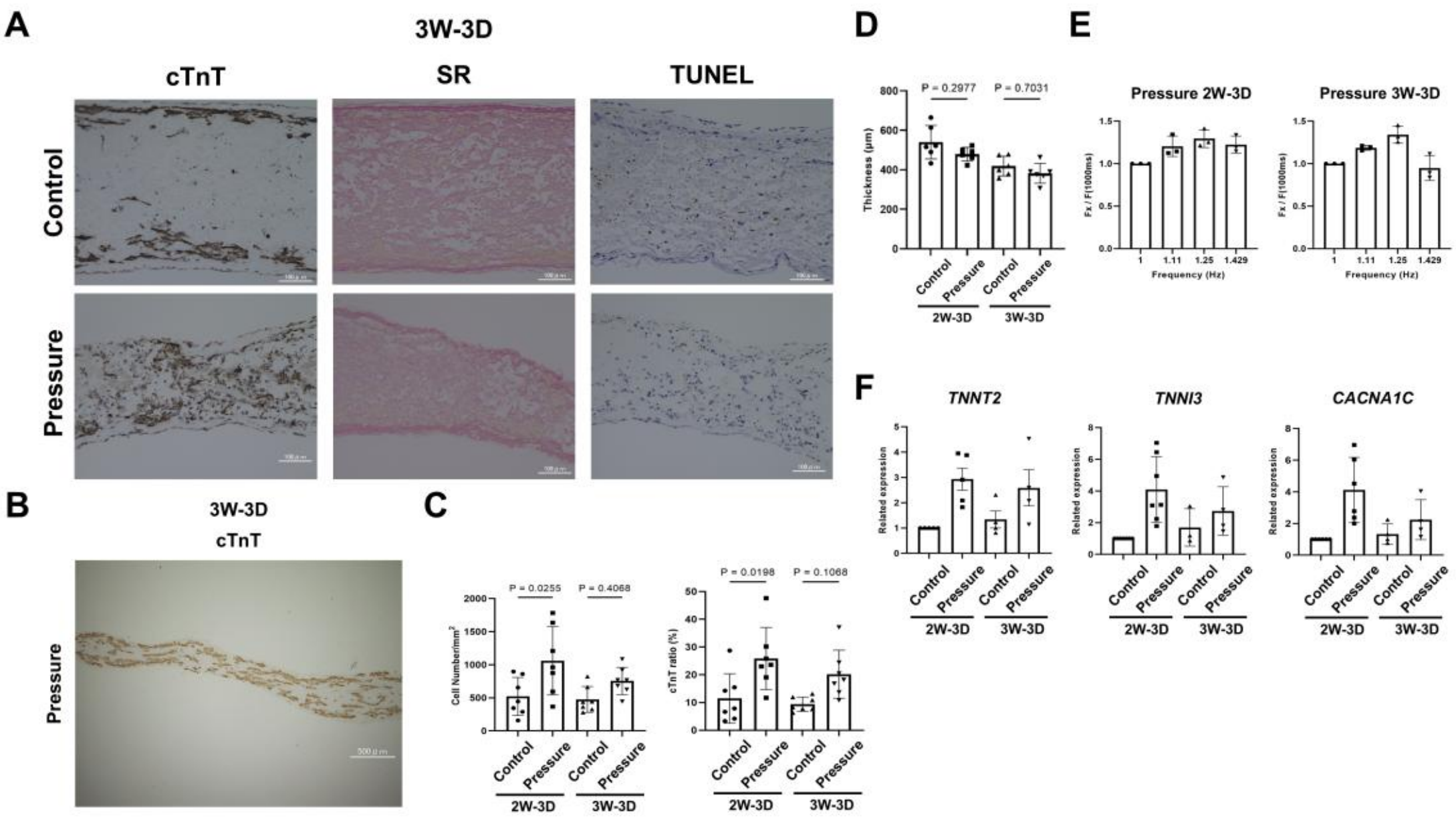

Figure S7: Intermittent training under different pre-maturation culture duration. (A) Representative immunostaining for cTnT (left), Sirius red staining (center), and TUNEL staining (right). 3W-3D: pre-maturation 3 weeks and stimulation 3 days. N = 3 independent ECTs derived from independent differentiation experiments. Scale bars = 100 μm. **(B)** Representative immunostaining for cTnT [lower magnification image of (A); left]. N = 3 independent ECTs derived from independent differentiation experiments. Scale bar = 500 μm. **(C)** Quantitative analysis for cell number (/mm^2) evaluated by hematoxylin and eosin staining (left) and cTnT positive ration among whole tissue area evaluated by cTnT immunostaining (right). N = 3 independent ECTs derived from independent differentiation experiments. **(D)** Quantification of ECT thickness width under 2W–3D and 3W–3D conditions. N = 6 independent ECTs derived from independent differentiation experiments. **(E)** Quantified changes in the motion amplitude in accordance with frequency of external electrical stimulation. Fx represents F(1000ms), F(900ms), F(800ms) and F(700ms), correspondingly. N = 3 independent ECTs derived from independent differentiation experiments. **(F)** RT-qPCR. TNNT2: cardiac troponin T2; TNNI3: troponin I3; CACNA1C: calcium voltage-gated channel subunit alpha1 C. N = 7 independent ECTs derived from 4 independent differentiation experiments.

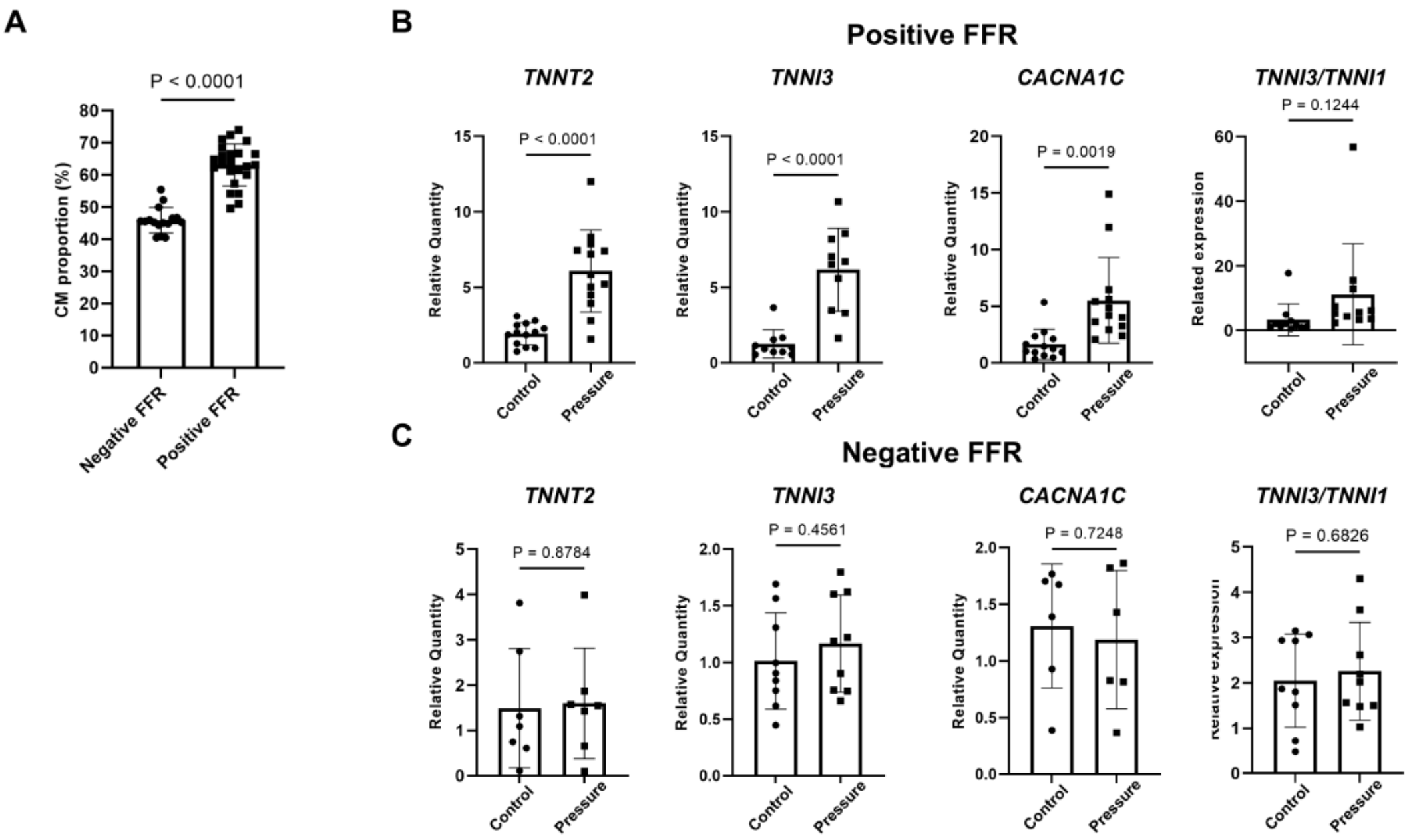

Figure S8: Force-frequency relationship (FFR) in accordance with the CM proportion of the ECTs. **(A)** CM proportion of ECTs. Positive FFR (n = 24) and Negative FFR (n = 16) independent samples. **(B)** RT-qPCR in ECTs with positive FFR. Positive FFR (n = 13, from 10 independent differentiation experiments) and Negative FFR (n = 9, from 6 independent differentiation experiments). **(C)** RT-qPCR in ECTs with negative FFR. Positive FFR (n = 13, from 10 independent differentiation experiments) and Negative FFR (n = 9, from 6 independent differentiation experiments).

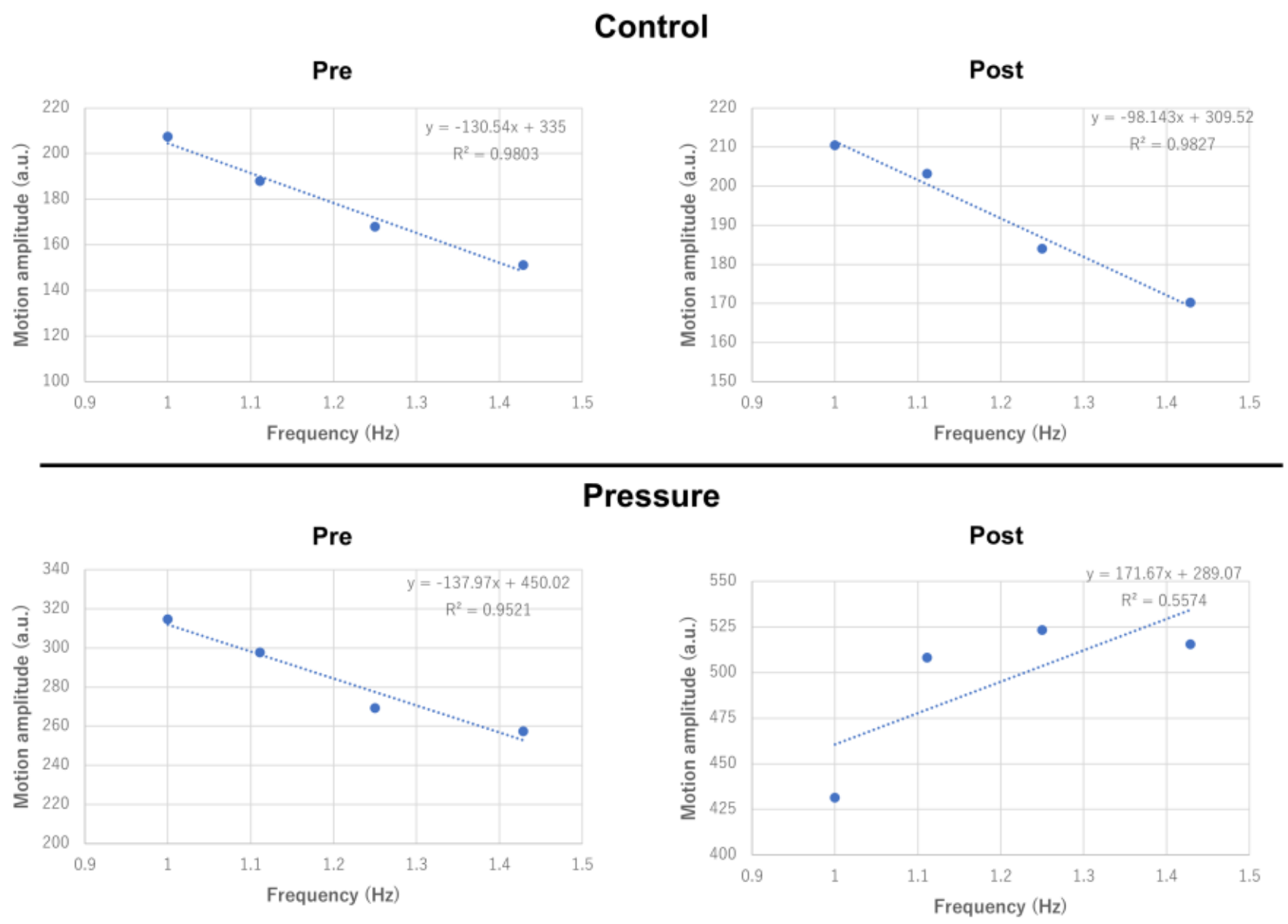

Figure S9: Representative relationship between the frequency of electrical stimulation frequency (X) and the motion amplitude (Y) in the whole beating cycle. This plot is associated with Figure 2B, C. Control (n = 12) and Pressure (n = 16) independent ECTs derived from independent differentiation experiments.

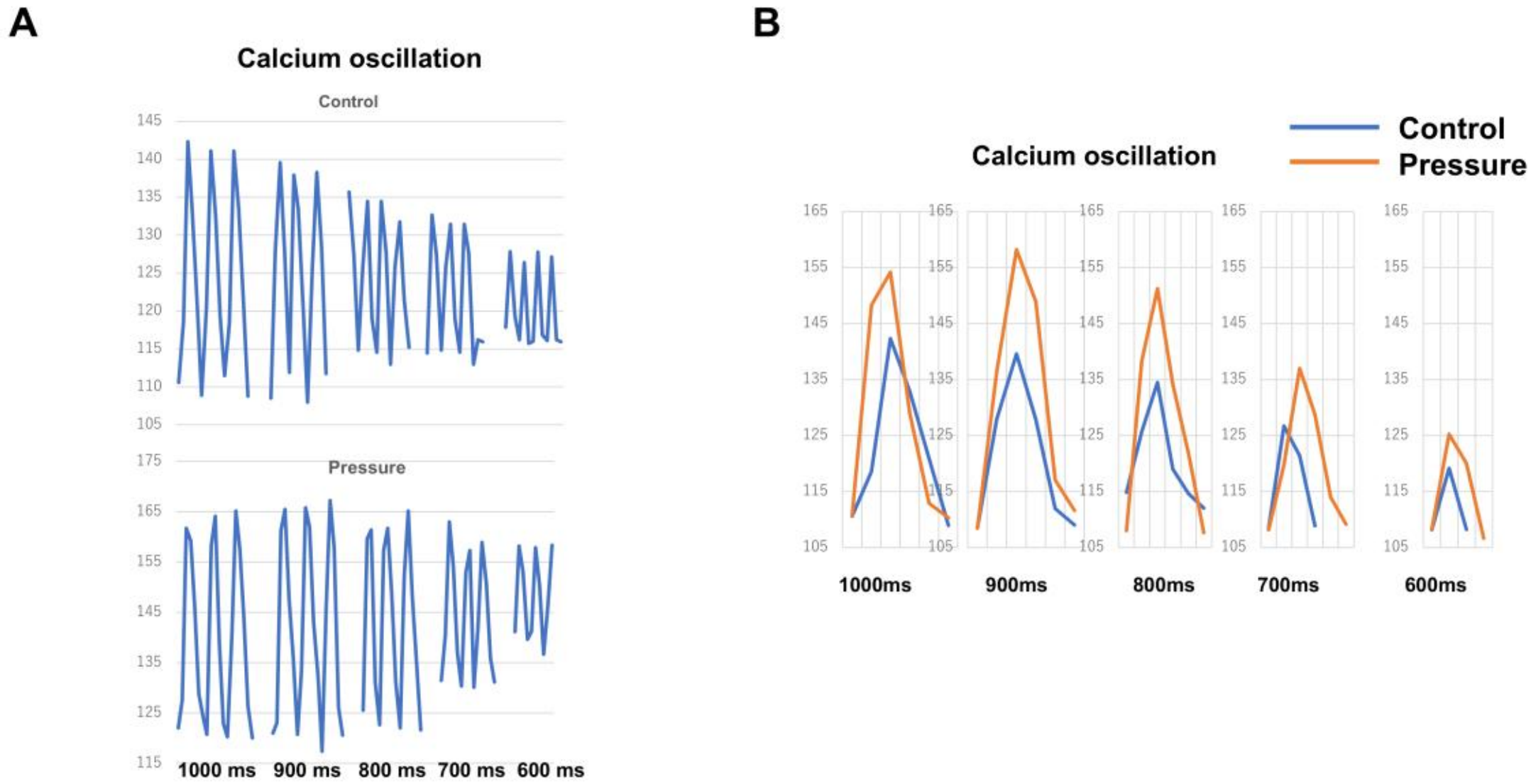

Figure S10: Calcium oscillation in accordance with external electrical pacing. (A) motion amplitude of calcium oscillation in accordance with frequency of external electrical stimulation. **(B)** Single cycle motion amplitude of calcium oscillation in accordance with frequency of external electrical stimulation. For (A) and (B), n = 10 independent ECTs derived from independent differentiation experiments.

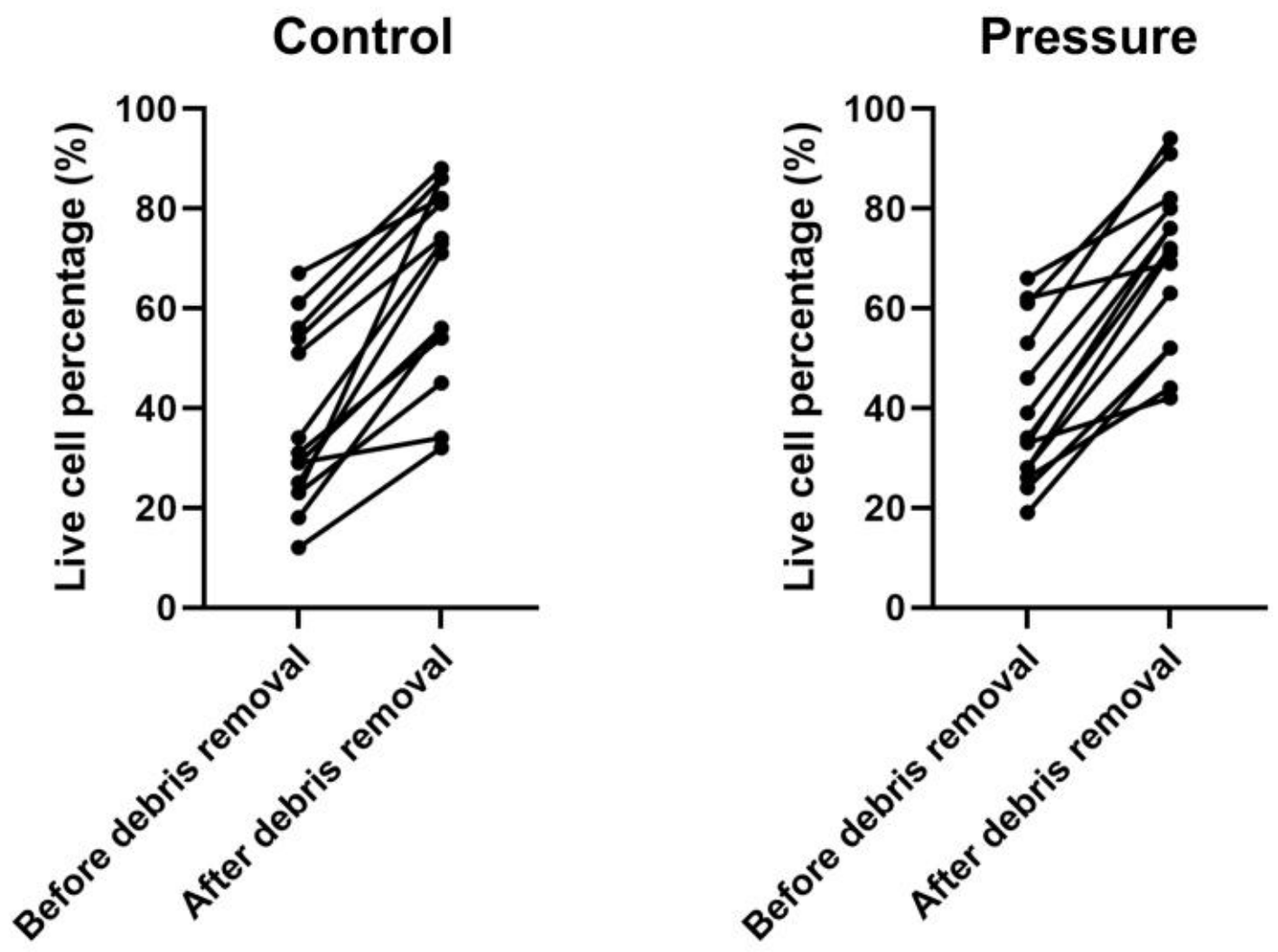

Figure S11: Live cell percentage of yielded cells after dissociation of ECTs, and before and after debris removal. N = 12 independent ECTs derived from independent differentiation experiments.

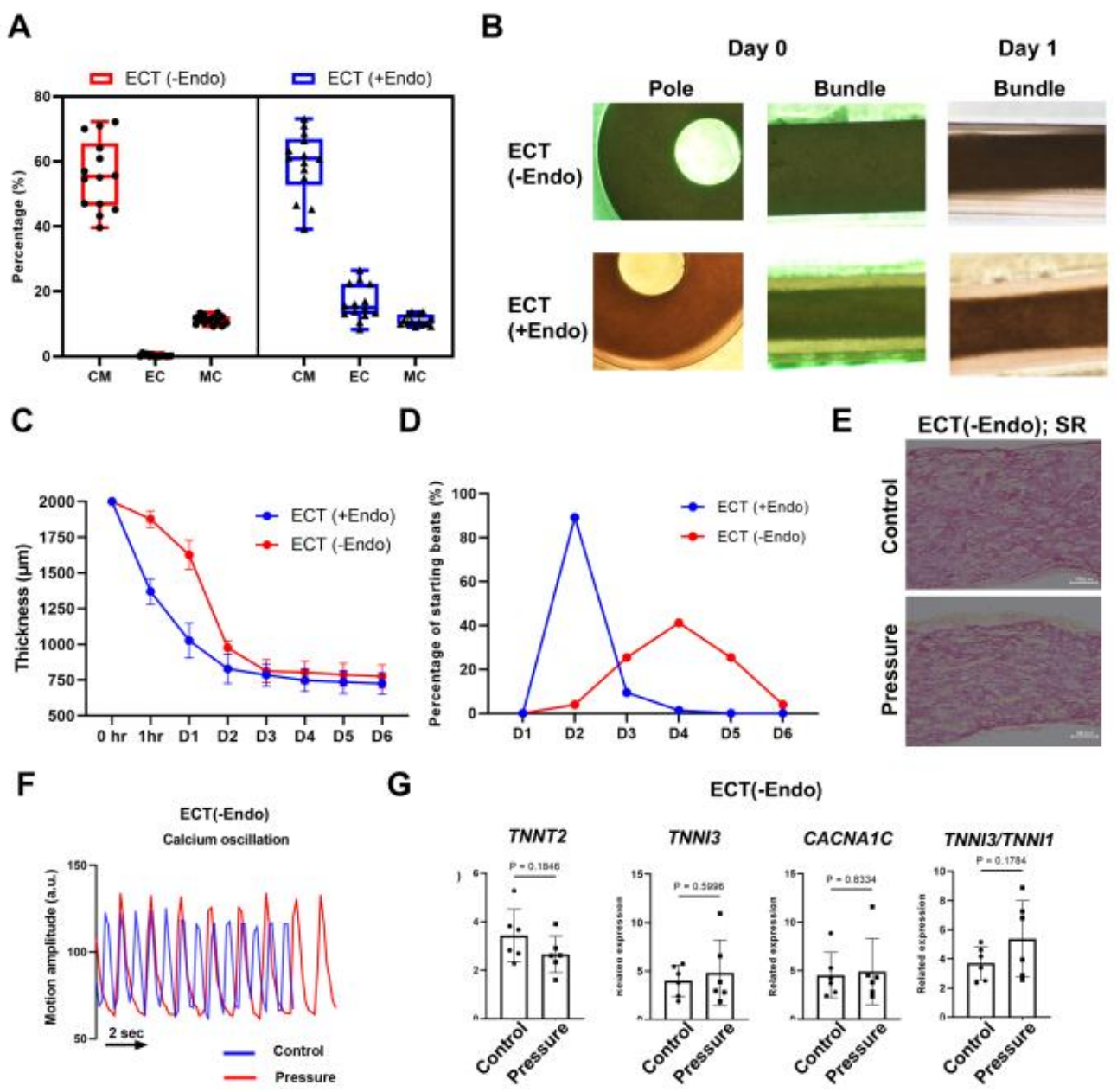

Figure S12: Structural and functional characteristics of ECTs without ECs [ECT (-Endo)]. **(A)** Cellular components of ECTs. ECT(-Endo): ECT without vascular endothelial cells; ECT(+Endo): ECT with vascular endothelial cells. N = 14 independent ECTs derived from independent differentiation experiments. **(B)** Microscopic observations at pole and bundle of ECTs on day 0 and 1. **(C)** Changes in the thickness of each type of ECTs over time after generation. **(D)** The timing of the initiation of beating of ECTs. **(E)** Representative SR stating of ECT(-Endo). Scale bars = 100 μm. **(F)** Representative calcium oscillation in spontaneous beating of ECT(-Endo). **(G)** RT-qPCR. For (B) to (D), n = 6 independent ECTs derived from independent differentiation experiments. For (E) to (G), n = 6 independent ECTs derived from independent differentiation experiments.

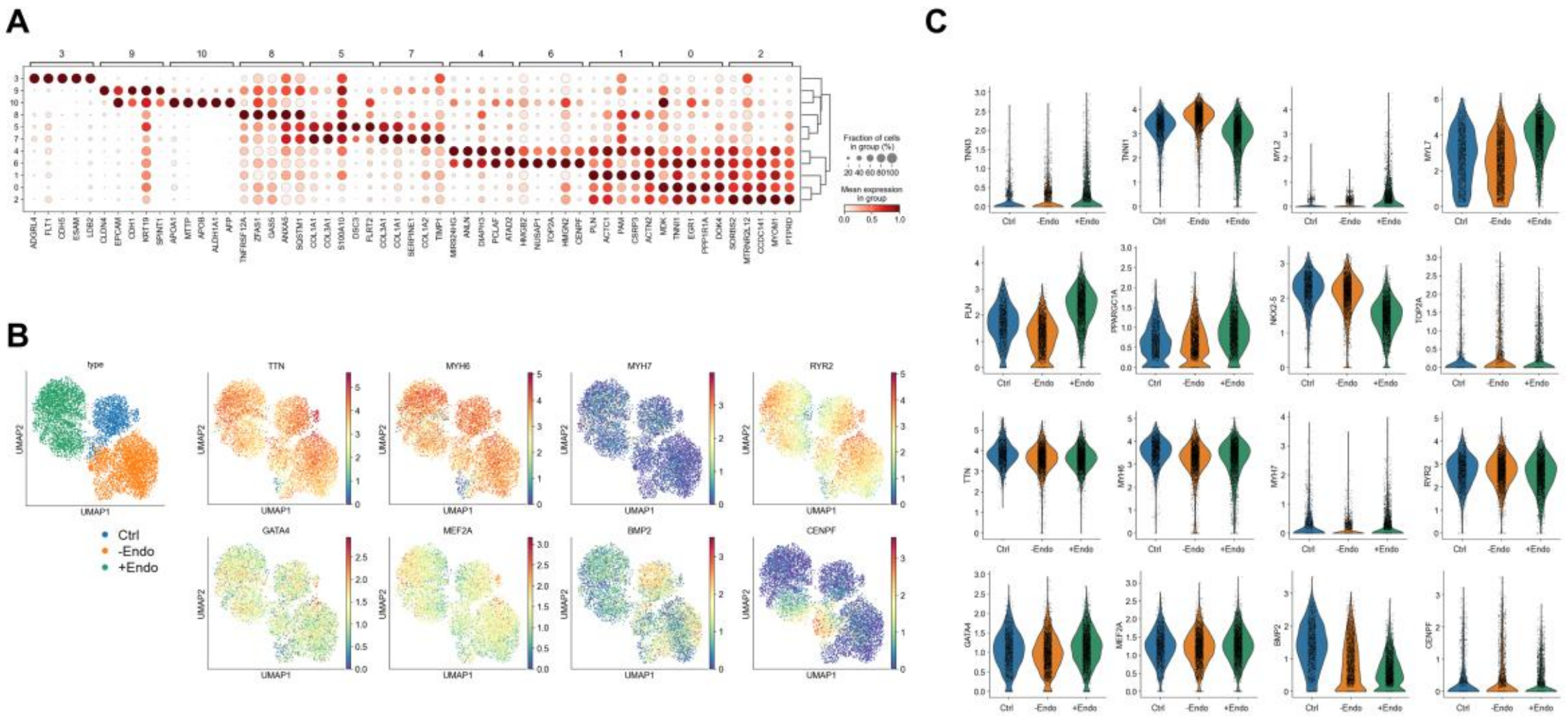

Figure S13: Single cell RNA-seq for total population and CM subpopulation. **(A)** Dot plot analysis for total population using marker genes for each cluster. **(B)** Feature plot analysis for CM subpopulation with maturation-related genes. **(C)** Violin plot analysis for CM subpopulation with maturation-related genes. For (A) to (C), n = 1 ECT per condition derived from 3 independent differentiation experiments.

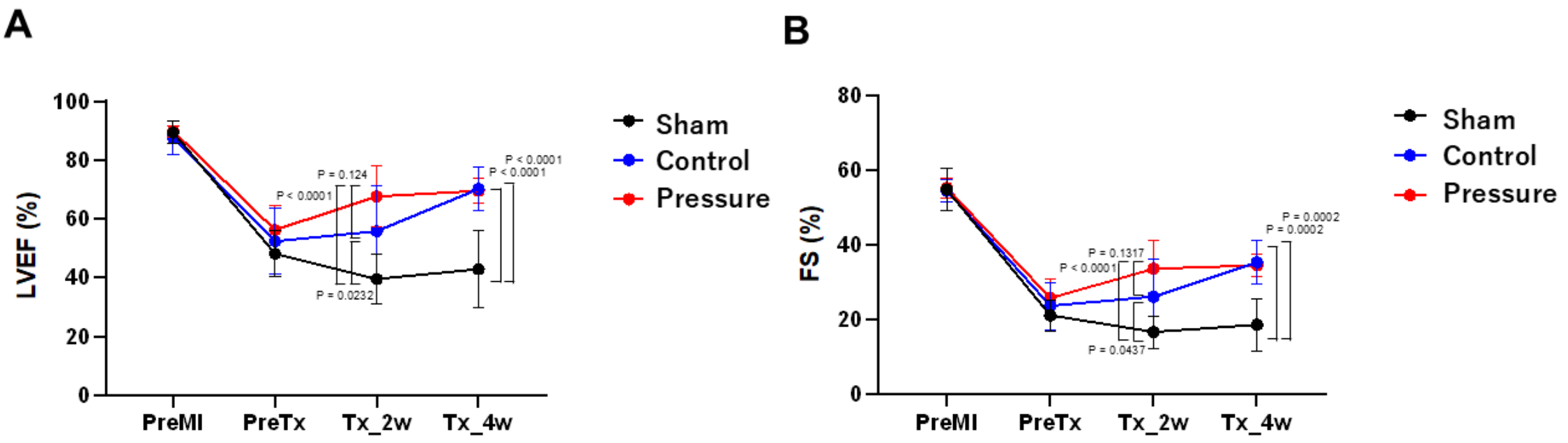

Figure S14: Temporal comparison of therapeutic effects following transplantation of pressure-trained and non-trained ECTs onto a rat myocardial infarction model. (A,B) Echocardiography. [n = 5 (Sham), 4 (Control; non-trained ECT) and 5 (Pressure; trained ECT)]**.** Pre MI: before induction of MI; PreTx: before treatment; Tx_2w/4w, 2/4 weeks after treatment. **(A)** Left ventricular ejection fraction (LVEF). **(B)** Fractional shorting (FS). For (A) and (B), Sham (n = 5), Control/non-trained (n = 4), Pressure/trained (n = 5) ECTs derived from independent differentiation experiments.

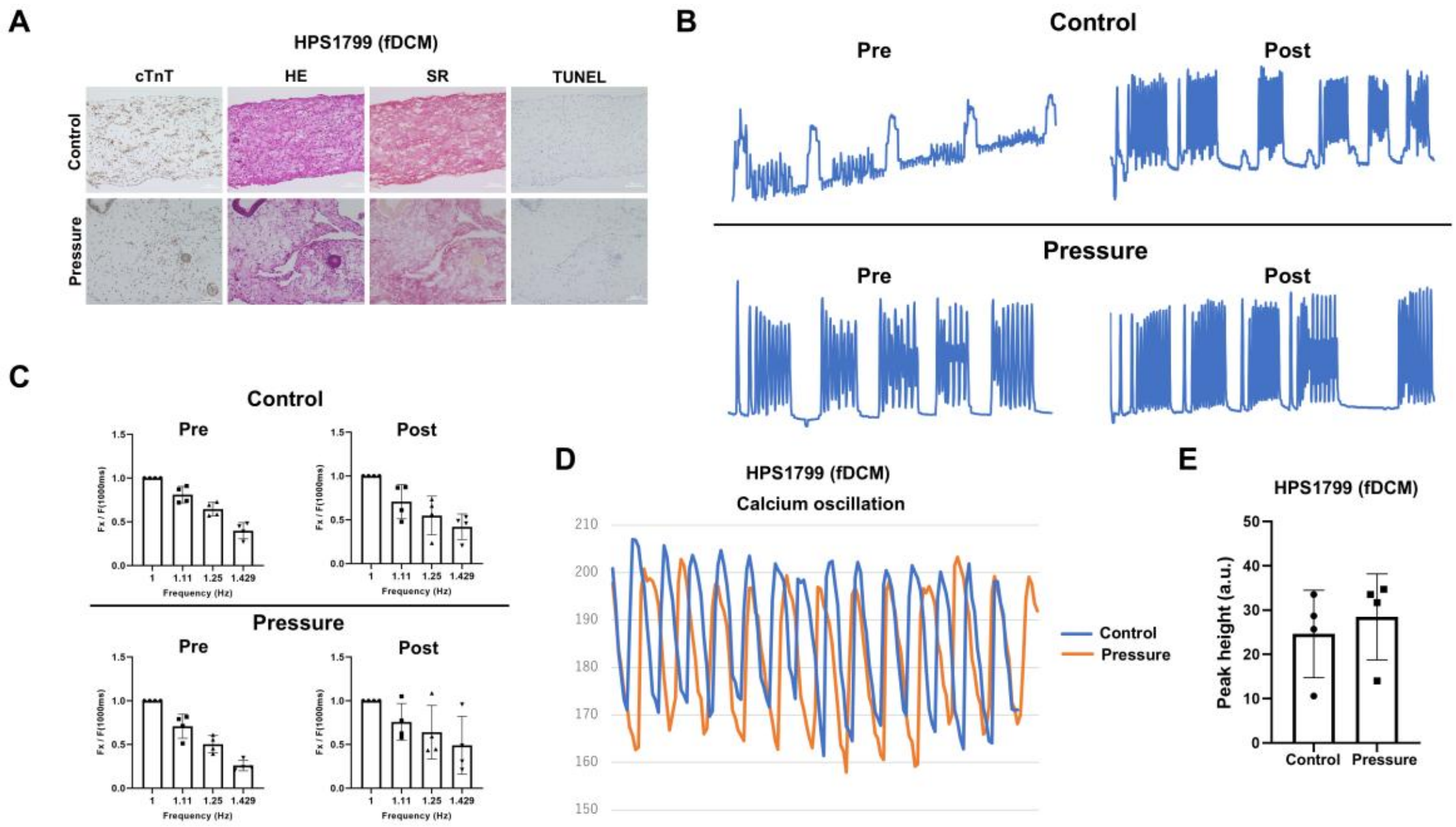

Figure S15: Hydrostatic pressure training results for familial dilated cardiomyopathy (fDCM) patient-derived ECTs. (A) Representative histological evaluations. HE: hematoxylin and eosin staining. N = 3 independent ECTs derived from independent differentiation experiments. Scale bars = 100 μm. **(B)** Representative waveforms and **(C)** quantified changes in the motion amplitude in accordance with the interval and frequency of external electrical stimulation before and after pressure training. **(D)** Representative calcium oscillation in spontaneous beating and **(E)** peak height. For (B) to (E), n = 4 independent ECTs derived from independent differentiation experiments.

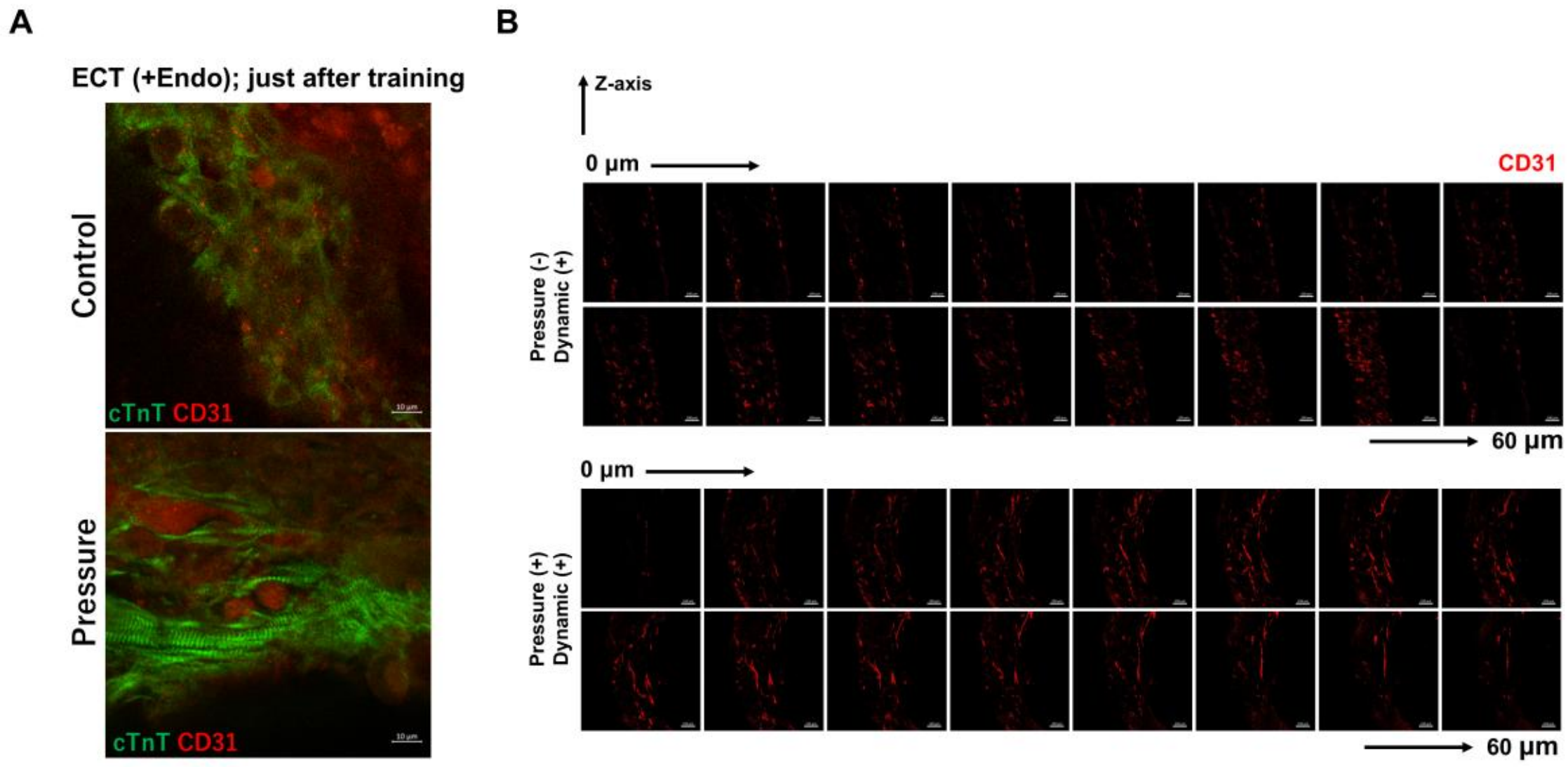

Figure S16: Confocal microscopic imaging for ECTs. **(A)** Confocal microscopic imaging for cTnT and CD31 immunofluorescent staining of ECT(+Endo) just after pressure training. **(B)** Cross sections of confocal microscopic imaging for CD31 immunofluorescent staining before constructing Z-stack image (Figure 6B).
